# RfaH Counter-Silences Inhibition of Transcript Elongation by H-NS–StpA Nucleoprotein Filaments in Pathogenic Escherichia coli

**DOI:** 10.1128/mbio.02662-22

**Published:** 2022-10-20

**Authors:** Christine M. Hustmyer, Michael B. Wolfe, Rodney A. Welch, Robert Landick

**Affiliations:** a Department of Biochemistry, University of Wisconsin–Madison, Madison, Wisconsin, USA; b Department of Medical Microbiology and Immunology, University of Wisconsin–Madison, Madison, Wisconsin, USA; c Department of Bacteriology, University of Wisconsin–Madison, Madison, Wisconsin, USA; Massachusetts Institute of Technology

**Keywords:** bacterial chromatin, ChIP-seq, counter-silencing, gene silencing, H-NS, RNAP, transcript elongation

## Abstract

Expression of virulence genes in pathogenic Escherichia coli is controlled in part by the transcription silencer H-NS and its paralogs (e.g., StpA), which sequester DNA in multi-kb nucleoprotein filaments to inhibit transcription initiation, elongation, or both. Some activators counter-silence initiation by displacing H-NS from promoters, but how H-NS inhibition of elongation is overcome is not understood. In uropathogenic E. coli (UPEC), elongation regulator RfaH aids expression of some H-NS-silenced pathogenicity operons (e.g., *hlyCABD* encoding hemolysin). RfaH associates with elongation complexes (ECs) via direct contacts to a transiently exposed, nontemplate DNA strand sequence called operon polarity suppressor (*ops*). RfaH–*ops* interactions establish long-lived RfaH–EC contacts that allow RfaH to recruit ribosomes to the nascent mRNA and to suppress transcriptional pausing and termination. Using ChIP-seq, we mapped the genome-scale distributions of RfaH, H-NS, StpA, RNA polymerase (RNAP), and σ^70^ in the UPEC strain CFT073. We identify eight RfaH-activated operons, all of which were bound by H-NS and StpA. Four are new additions to the RfaH regulon. Deletion of RfaH caused premature termination, whereas deletion of H-NS and StpA allowed elongation without RfaH. Thus, RfaH is an elongation counter-silencer of H-NS. Consistent with elongation counter-silencing, deletion of StpA alone decreased the effect of RfaH. StpA increases DNA bridging, which inhibits transcript elongation via topological constraints on RNAP. Residual RfaH effect when both H-NS and StpA were deleted was attributable to targeting of RfaH-regulated operons by a minor H-NS paralog, Hfp. These operons have evolved higher levels of H-NS–binding features, explaining minor-paralog targeting.

## INTRODUCTION

Horizontal gene transfer in bacteria provides genetic diversity by spreading DNA from one organism to another (1). Transferred DNAs can be toxic to bacteria if expressed improperly, but also can provide new functions (2). For example, mobile AT-rich pathogenicity operons allow pathogenic Escherichia coli and related bacteria to infect and even kill humans (3–6). Bacteria silence foreign DNA using proteins like H-NS (histone-like nucleoid structuring protein), a bacterial chromatin protein that silences AT-rich DNA in E. coli (7–12).

**FIG 1:**
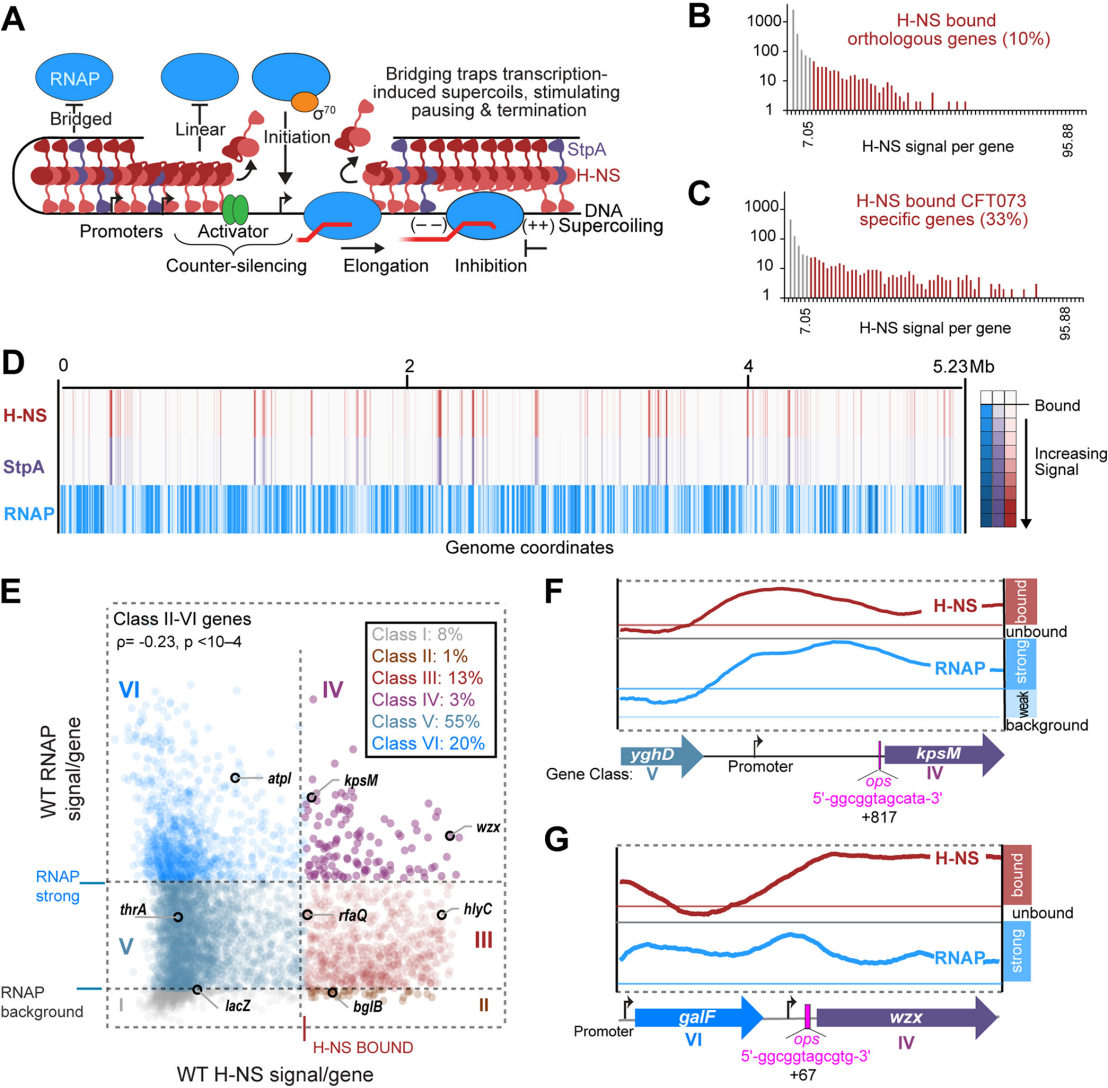
RNAP is excluded from many, but not all H-NS–StpA-bound genes. (A) H-NS effects on RNAP initiation and elongation. H-NS (dark and light red monomers) and StpA (purple) form linear and bridged filaments on DNA that prevent RNAP–σ^70^ (blue–orange) binding to promoters (black arrows). DNA-binding proteins (green), known as H-NS counter-silencers, can displace H-NS, allowing RNAP to initiate transcription. Only bridged, not linear, filaments inhibit transcript elongation by stimulating RNAP pausing and termination. (B) Histogram of H-NS ChIP signals on CFT073 protein-coding genes with orthologs in MG1655 (Data set S1A). Red and gray bars correspond to genes bound or not bound by H-NS, respectively. (C) Histogram of H-NS ChIP signals on CFT073 lineage-specific genes colored as for panel B (Data set S1A). (D) Heat map of H-NS (red), StpA (purple), and RNAP (blue) ChIP/Input signals in 1.4 kb windows across the CFT073 genome. Data are averages of three biological replicates. (E) Scatterplot of average (log_10_ scaled) RNAP ChIP/Input signal per gene versus average H-NS ChIP/Input signal per gene in CFT073 divided into 6 classes. Class boundaries are denoted by gray dashed lines. Black outline highlights genes of interest. Data are averages of three biological replicates. (F) H-NS (red) and RNAP (blue) ChIP profiles around *c3698 (kpsM)*, a gene bound by both RNAP and H-NS (class IV). Genes are color coded based on RNAP–H-NS relationship class. Red box in H-NS track represents H-NS bound cutoff and the dark blue box indicates strong RNAP cutoff, while light blue box represents weak RNAP signal. RfaH *ops* motif indicated by magenta bar. Transcription starts sites (TSSs) (black arrows) and predicted promoters were identified using RNA-seq data (73). Data are averages of 3 biological replicates. (G) H-NS and RNAP around *wzx*, a second class IV gene presented similarly to *kpsM* (panel F).

H-NS forms long nucleoprotein filaments on DNA (0.2–20 kb) (13, 14). These filaments can block RNA polymerase (RNAP) initiation (15), prevent promoter escape (16), and impede RNAP elongation by promoting transcriptional pausing and trapping RNAP topologically (17). H-NS oligomers bind either one DNA duplex (linear filaments, also called stiffened or hemi-sequestered) or two segments of DNA duplex (bridged filaments) (17–20). Both linear and bridged filaments can inhibit transcription initiation by blocking promoters. Bridged but not linear filaments promote pausing (and ρ-dependent termination) by trapping RNAP in topologically closed domains that impede forward translocation and promote backtracking (Fig. 1A) (17). However, the distribution of linear versus bridged filaments *in vivo* is unknown.

Many enterobacteria contain multiple H-NS paralogs (e.g., StpA) and modulating proteins that bind H-NS filaments (e.g., Hha) (21, 22). These paralogs and modulators may alter H-NS silencing by affecting H-NS bridging (19, 22–25). StpA (58% identical to H-NS) forms heterodimers with H-NS (26), promotes bridging, and increases RNAP pausing (Fig. 1A) (23). Hha binds the N-terminal domain of H-NS, cannot bind DNA alone, and enhances bridging and RNAP pausing (19, 23, 27).

H-NS binds ~15% of the E. coli genome (9, 10). StpA, present at about 1/4th the level of H-NS (23, 28), exhibits the same genomic distribution as H-NS in K-12 and enterohemorrhagic E. coli (29, 30). Deletion of *hns* mostly activates gene expression (24, 31, 32). StpA can partially compensate for loss of H-NS (11, 24, 33–35), potentially by binding to certain high affinity sites (29, 36). Deletion of *stpA* alone has no reported phenotype in E. coli (29, 37). However, deletion of both *hns* and *stpA* cripples cell growth and alters transcription genome-wide (37). Uropathogenic E. coli (UPEC) encodes additional H-NS paralogs and modifiers not found in K-12, including the paralog Hfp (58% identical to H-NS) (33, 38). Deletion of *hns* increases Hfp expression levels 5-fold (33). However, distributions of H-NS, StpA, and Hfp in UPEC, and the synergistic mechanisms by which these proteins regulate silencing, are unknown.

Bacteria counter H-NS silencing of transcription initiation using DNA-binding activators that bind near promoters and displace or rearrange H-NS in a mechanism called counter-silencing (Fig. 1A) (39–42). However, it is unclear how RNAP overcomes H-NS silencing during transcript elongation. The elongation regulator RfaH, which aids elongation through bridged H-NS *in vitro* (17), may counter H-NS silencing *in vivo*. RfaH is a specialized paralog of NusG, the only transcription factor conserved in all domains of life (43, 44). RfaH inhibits RNAP pausing and termination, recruits ribosomes that may aid transcript elongation, and excludes NusG (45–47). Since NusG aids ρ, RfaH also indirectly inhibits ρ-dependent termination (48, 49). RfaH is recruited to two operons in E. coli K-12 (47) via a specific 12-nt sequence called operon polarity suppressor (*ops*), when *ops* is exposed in the nontemplate DNA strand of paused transcription complexes (Fig. S2A) (50). Although RfaH positively regulates virulence operons (51–54), neither the distribution of RfaH in UPEC, nor its direct relationship to H-NS in controlling gene expression and RNAP occupancy, is known.

**FIG 2:**
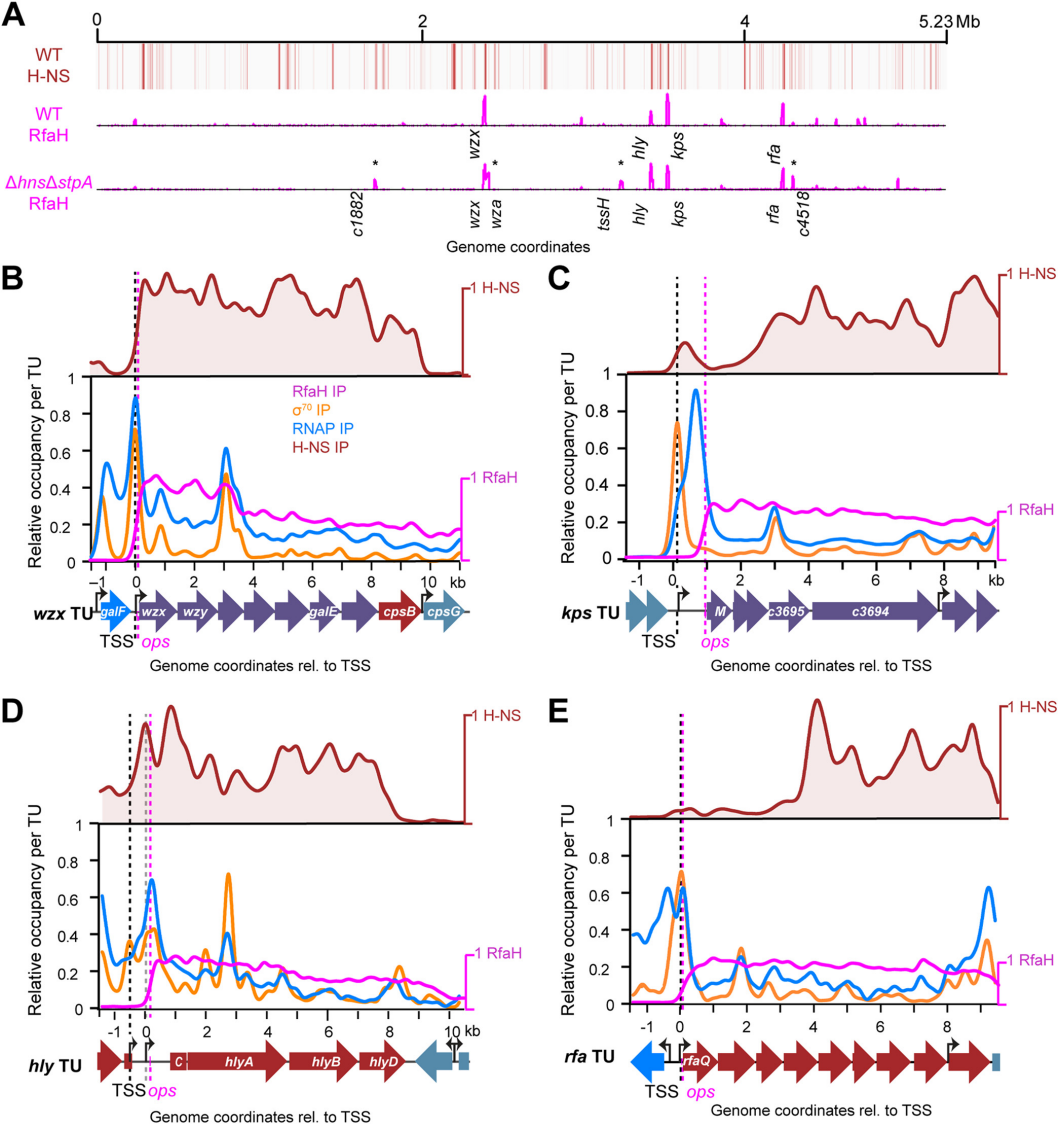
RfaH targets four H-NS bound loci in WT CFT073. (A) Genome-scale heat map of H-NS ChIP signal in CFT073 (red) compared to RfaH ChIP signal profiles for WT and Δ*hns*Δ*stpA* CFT073 (magenta peaks; see Data set 2). RfaH-bound loci revealed by deletion of *hns* and *stpA* are marked with an asterisk. (B) ChIP signals for H-NS (red), σ^70^ (orange), RNAP (blue), and RfaH (magenta) scaled to local maximal and minimal signals for each target (Methods). Black arrows and dashed black line indicate predicted TSSs, dashed magenta line indicates location of *ops* site. Traces are averages of two (RfaH) or three (others) replicates. (C), (D), (E) same as (A) but for *kps*, *hly*, and *rfa* loci, respectively. Black and gray dashed lines in (C) indicate promoters predicted from E. coli ST131 5′ RACE or CFT073 RNA-seq data, respectively (73, 74). Single letter gene labels *M* and *C* correspond to *kpsM* and *hlyC*, respectively.

10.1128/mbio.02662-22.2 FIG S2 *ops* motif locations in CFT073. (A) Mechanism of RfaH recruitment to transcribing RNAP via *ops*. RfaH (magenta) and σ^70^ (orange) bind to overlapping sites on RNAP (blue). Free RfaH is autoinhibited, but remodels once recruited to RNAP paused at an *ops* sequence by interactions with a nontemplate DNA hairpin encoded by *ops* (yellow hairpin). RfaH promotes RNAP elongation and excludes NusG (green), thus inhibiting ρ-dependent termination (red stop sign) and recruiting a ribosome (white) to enable transcription–translation coupling. (B) Scatter plot (log_10_-scaled) of average RNAP occupancy, scaled to global maxima and minima (Methods) per gene in WT vs. Δ*hns*Δ*stpA* CFT073 strains. Data are averages of at least 2 biological replicates. (C) *ops* sequence logo generated from *ops* motifs in the eight identified RfaH-bound loci in CFT073 (Data set 2). (D) *ops* sequence locations in CFT073 genome-wide based on match to sequence logo (Data set 2B, E). T and NT gene body, *ops* located on the template or nontemplate strand of coding regions, respectively. (E) Overlay of WT RfaH IP/Input signal (magenta) and Δ*hns*Δ*stpA* RfaH IP/Input signal (dark purple) at *wzx* loci (see Fig. 2). Traces represent averages of 2 biological replicates (F), (G), (H) Same as (E) but for *kps*, *hly*, or *rfa* loci, respectively. (I) Genome-scale view of RfaH ChIP profiles in WT (magenta) and Δ*rfaH* strains (dark magenta). Gray dashed boxes indicate the 4 RfaH-bound RNAP loci in CFT073 in WT conditions. Gray-boxed blowup show the RfaH ChIP signal profiles for WT and Δ*rfaH* CFT073 at the *wzx* locus. See Dataset S2 for RfaH loci distribution. Download FIG S2, TIF file, 1.9 MB. Copyright © 2022 Hustmyer et al. 2022 Hustmyer et al. https://creativecommons.org/licenses/by/4.0/ This content is distributed under the terms of the Creative Commons Attribution 4.0 International license.

To gain direct insight into H-NS and RfaH function, we used ChIP-seq to map H-NS, StpA, RNAP, σ^70^, and RfaH in six derivatives of UPEC strain CFT073: wild-type (WT), Δ*rfaH*, Δ*stpA*, Δ*stpA*Δ*rfaH*, Δ*hns*Δ*stpA*, and Δ*hns*Δ*stpA*Δ*rfaH.* Our experiments map transcription and gene silencing in CFT073, identify new RfaH-regulated loci in CFT073, and reveal that RfaH acts as an elongation counter-silencer of H-NS–StpA gene silencing at crucial pathogenicity loci.

## RESULTS

### H-NS–StpA and RNAP are anti-correlated on most but not all CFT073 genes.

To determine which CFT073 genes are bound by H-NS, we used ChIP-seq with polyclonal anti-H-NS antibodies. We chose polyclonal antibodies because epitope tags potentially perturb DNA-binding and bridging (19, 20). To ask if H-NS preferentially binds lineage-specific (i.e., horizontally acquired) genes, we compared H-NS–bound genes in WT E. coli K-12 and CFT073 using the average H-NS signal per gene (Data set S1A). We used an inflection point in the signal distribution to define H-NS–bound genes (Fig. S1A and B). We determined most K-12 and CFT073 (~77%) genes are orthologs (Data set S1A). CFT073 orthologous genes bound H-NS infrequently (~10% bound; Fig. 1B and Data set S1B and C) compared to lineage-specific genes, of which ~33% bound H-NS (Fig. 1C). The lineage-specific, H-NS bound genes included RfaH-regulated genes in the hemolysin (*hly*) and polysaccharide capsule (*kps*) operons (52, 55, 56). We conclude that H-NS preferentially binds lineage-specific genes in K-12 and CFT073, consistent with the hypothesis that H-NS functions in part to silence horizontally transferred genes.

10.1128/mbio.02662-22.1 FIG S1 H-NS and StpA are similarly distributed in UPEC and K-12. (A) Histograms of average H-NS/Input signal for coding genes in CFT073 (bottom, Data set S1). Cumulative number of bound genes at increasing H-NS ChIP signal (top). Red bars and points indicate H-NS bound genes. Data are averages of three biological replicates. The cutoff used for designation as H-NS bound is indicated by the dashed red line. (B) Same as (A), but for E. coli K-12 strain RL3000 from a single replicate. (C) Heat map of H-NS (red) and StpA (purple) ChIP signals in 1.4 kb windows across the CFT073 genome in WT, Δ*stpA*, and Δ*hns* strains (Methods, Text S1). Data represent averages of at least 2 biological replicates. (D) Scatter plot of log_10_-scaled average StpA vs. H-NS ChIP signal per gene in CFT073. Red and purple dotted lines indicate signal for *atpB*, which is scored as not bound by H-NS or StpA. Data are averages of three biological replicates. Spearman correlation coefficients are reported. (E) Scatter plot of H-NS and StpA signal per gene in MG1655 using ChIP-exo raw data deposited under GEO GSE181767. In this dataset, both StpA and H-NS were C-terminal Myc-tagged and harvested in mid-log phase in M9 minimal media with 0.2% glucose. Read coverage was determined at 5 bp resolution, scaled by the median read count over all bins, and plotted per gene. X-axis is log_10_ H-NS signal/gene and Y-axis is log_10_ StpA signal/gene. Red and purple dotted line indicates the average signal at non-H-NS bound gene *atpB* to symbolize background in the H-NS and StpA signals respectively. Data is the average of 2 biological replicates and the Spearman correlation coefficient is noted on graph. (F) Western blot using pre-cleared H-NS antiserum to assess specificity of antibody in CFT073. CFT073 cells were collected in WT, Δ*hns*, Δ*stpA*, and Δ*hns*Δ*stpA* at an OD_600_=0.4 in MOPS rich defined media to reflect ChIP-seq conditions. Concentrations of each lysate were titrated on the gel. (G) Same as E, but with StpA polyclonal antiserum. Lysates in (E) were the same lysates used in (F). Download FIG S1, TIF file, 2.8 MB. Copyright © 2022 Hustmyer et al. 2022 Hustmyer et al. https://creativecommons.org/licenses/by/4.0/ This content is distributed under the terms of the Creative Commons Attribution 4.0 International license.

10.1128/mbio.02662-22.8 Data set S1 (A) Per gene class analysis of RNAP and H-NS in CFT073 (B) Summary of genome-wide H-NS binding in CFT073 and MG1655 (C) H-NS signal per gene in MG1655 (D) RNAP and H-NS class analysis summary (E) H-NS and StpA signal on genes in CFT073 (F) Summary of H-NS and StpA signal on genes on all strains (G) DNA sequence discrepancies with NCBI record NC_004431.1 for CFT073 data sets (H) Sequence differences found in only a subset of CFT073 datasets. Download Data Set S1, XLSX file, 0.8 MB. Copyright © 2022 Hustmyer et al. 2022 Hustmyer et al. https://creativecommons.org/licenses/by/4.0/ This content is distributed under the terms of the Creative Commons Attribution 4.0 International license.

Next, we asked how the bridging enhancer StpA is distributed within H-NS filaments genome-wide in CFT073 and K-12. For StpA, we generated polyclonal antibodies against purified StpA expressed in a Δ*hns* strain and observed minimal cross-reactivity of H-NS and StpA antibodies by Western blot (Fig. S1F and G). StpA was distributed almost indistinguishably from H-NS in CFT073 (Fig. 1D). The strong antibody preference for its cognate paralog and the near-identical ChIP patterns obtained when both H-NS and StpA are present indicate uniform distribution of StpA within H-NS filaments genome-wide in CFT073 (Fig. S1C and D). We also analyzed published epitope-tagged H-NS and StpA ChIP-exo data for K-12 (57) and found that epitope-tagged H-NS and StpA are similarly distributed in K-12 (Fig. S1E), consistent with a ChIP-chip study of K-12 (29).

To ask if the H-NS–StpA–bound CFT073 genes were transcriptionally silenced, we next compared the distributions of H-NS and RNAP on protein-coding genes by ChIP-seq (Fig. 1E). Prior analyses of E. coli K-12 suggest RNAP and H-NS may co-localize (8, 58). However, these analyses included promoter regions where H-NS and RNAP may be bound at adjacent sites too close to distinguish by ChIP-chip. We focused on coding regions where fewer promoters are located and calculated average per gene occupancies of H-NS and RNAP. We divided genes into 6 classes based on being H-NS–bound or unbound and exhibiting either low, medium, or high RNAP occupancy corresponding to no transcription (indistinguishable from nonspecific background association of non-transcribing RNAP, e.g., *lacZ*), moderate transcription (most genes, e.g., *thrA*) or high transcription (e.g., *atpI*) (59, 60) (Fig. 1E and Data set S1A). H-NS and RNAP occupancy were generally anticorrelated, consistent with H-NS silencing elongation. Many H-NS–bound genes exhibit weak RNAP signal (class III [Fig. 1E]). These low levels of RNAP may reflect cryptic internal promoters common in many H-NS silenced genes (2, 15, 61–63).

Of particular interest, only ~3% of CFT073 genes exhibited both H-NS binding and strong RNAP binding (class IV [Fig. 1E]). Among these genes were several in operons known to be targeted by RfaH in non-UPEC enterics (e.g., *kps* [capsule synthesis] and *wzx* [colonic acid and O-antigen synthesis]) (47, 64, 65). We identified *ops* motifs in the 5′-leader regions of *kps* and *wzx* in CFT073 (Fig. 1F and G). High RNAP signal despite high H-NS signal at these known RfaH targets suggests RfaH could function as an elongation counter-silencer of H-NS-mediated gene silencing. Two other known RfaH targets, the hemolysin (*hly*) and *rfaQ*/*waaG* lipopolysaccharide synthesis operons (47, 51, 66), were also bound by H-NS but exhibited lower levels of RNAP and thus were in class III (Fig. 1E).

### RfaH targets eight CFT073 loci, all bound by H-NS and four also silenced for initiation.

To ask if RfaH regulation depends on H-NS inhibition of RNAP elongation, we mapped RfaH-bound ECs in CFT073 by ChIP-seq using anti-RfaH polyclonal antibodies. RfaH associates with DNA only via its interaction with elongating RNAP at or downstream from an *ops* site (Fig. S2A). In WT CFT073, RfaH associated with RNAP at 4 loci (*wzx*, *hly*, *kps*, and *rfa*), all of which were also coated by H-NS–StpA filaments (Fig. 2A and Data set S2A). Consistent with prior findings in E. coli K-12 (45, 67), RfaH remained bound to RNAP throughout transcription units (TUs) downstream from the single *ops* present in each TU. No *ops* sites were identified outside the leader regions, making RfaH reloading unlikely (Fig. 2B to E and Data set S2B). We focused analysis on RfaH signal at predicted TUs, although RfaH-ECs continue transcription downstream of predicted TUs in some cases (Fig. S2E to H). The signal(s) that terminate RfaH-modified ECs are currently unclear and an interesting topic for future research.

10.1128/mbio.02662-22.9 Data set S2 (A) Description of TUs with RfaH signal in CFT073 (B) FIMO catalog of *ops* locations genome-wide and corresponding RfaH and RNAP signal (C) WT RfaH signal peak calling (D) *ΔhnsΔstpA* RfaH signal peak calling (E) RNAP signal at predicted *ops* sites genome-wide. Download Data Set S2, XLSX file, 0.03 MB. Copyright © 2022 Hustmyer et al. 2022 Hustmyer et al. https://creativecommons.org/licenses/by/4.0/ This content is distributed under the terms of the Creative Commons Attribution 4.0 International license.

We next asked if the RfaH and RNAP distributions change in the absence of H-NS–StpA filaments. ChIP-seq revealed large-scale changes in RNAP locations in Δ*hns*Δ*stpA* versus WT CFT073 (Fig. S2B), consistent with H-NS silencing initiation and elongation (2, 61). RfaH remained associated with *wzx*, *kps*, *hly*, and *rfa* in Δ*hns*Δ*stpA* CFT073 (Fig. 2A and Fig. S2E to H) but was also associated with 4 previously uncharacterized loci (Data set S2A). Three loci (*c3392*–*tssH* encoding Type VI secretion and uncharacterized proteins; *c4518–c4511* encoding a putative ShlB family hemolysin secretion protein, a putative ImpA membrane protein, and uncharacterized proteins; and *c1882–c1889* encoding putative type VI secretion proteins and uncharacterized proteins) are distal from other RfaH-bound loci. Another, *wza–wcaM* encoding lipopolysaccharide synthesis enzymes, is directly upstream of *wzx*. All 4 new loci were bound proximally by H-NS and appeared to be inhibited for transcription initiation by H-NS–StpA in WT CFT073 grown in our conditions (rich medium) (Fig. 2A to E). *wza*, *tssH*, and *c1458* were in WT class I, indicating RNAP signal below background; *c4518* was in class V, indicating low RNAP signal in WT, although the signal is borderline (average RNAP signal, 0.44; background cutoff, 0.42). We conclude that *c3392*–*tssH*, *c4518–c4511*, *wza-wcaM*, and *c1882–c1889*, are likely cryptic RfaH-regulated loci that may require a regulatory initiation signal to counter H-NS–StpA promoter silencing.

To ask if all transcribed *ops* sites bind RfaH, we cataloged *ops* sites in CFT073 globally (Fig. S2C and Data set S2B). Of 42 potential *ops* sites genome-wide, only the 8 that generated RfaH ChIP signals, *wzx*, *hly*, *kps*, *rfa*, *wza*, *tssH*, *c4518*, and *c1882*, were in the nontemplate leader regions of operons (Fig. S2D and Data set S2B). RfaH ChIP signal was absent at these 8 sites in Δ*rfaH* (Fig. S2I and Data set S2C and D). The remaining 34 *ops* sites were within coding regions of annotated genes (Data set S2B and E); 22 were on the nontemplate strand, a requirement for functional RfaH recruitment to elongating RNAP (50, 68). Of these 22 genes, 77% had RNAP signal above background (Class II-VI), but all lacked RfaH signal (Data set S2E). RfaH binding at these sites may be excluded by NusG bound to coupled transcription–translation complexes (69–72). We conclude that CFT073 encodes 8 bona fide RfaH-regulated loci all bound by H-NS, supporting the RfaH counter-silencing model.

### σ^70^ ChIP-seq confirms RfaH associates downstream from predicted promoters.

To investigate RfaH association with RNAP relative to promoters, we generated σ^70^ ChIP-seq data for CFT073. We predicted transcription units (TUs) and transcription start sites (TSSs) using published CFT073 RNA-seq data (73) and compared predicted TSSs to σ^70^ ChIP-seq. For *wzx*, *kps*, and *rfa*, σ^70^ peaks were evident at predicted TSSs upstream from *ops* (Fig. 2B, D to E, black dotted lines). Consistent with H-NS affecting only elongation on these genes, H-NS ChIP signal did not extend over the predicted TSSs. For *hly*, we found two promoters upstream of the *ops* site. One, ~0.77 kb upstream from *hlyA*, was within the H-NS-repressed region (Fig. 2D, gray dotted line). The second, ~1.6 kb upstream from *hlyA* where H-NS signal was lower (Fig. 2D, black dotted line), was described previously (74).

The *wza*, *c4518*, and *c1882* loci, which showed evident RfaH association in the Δ*hns*Δ*stpA* strain, had apparent σ^70^ peaks upstream from each gene in Δ*hns*Δ*stpA* but not WT CFT073 (Fig. S3A to C). H-NS bound these regions in WT CFT073, confirming that H-NS ordinarily represses initiation at these loci (Fig. S3A to C). H-NS was not bound to the potential promoter regions of *tssH* but was bound internally and upstream (Fig. S3D), suggesting transcription initiation at *tssH* is indirectly controlled by H-NS–StpA. For example, initiation may rely on an activator whose expression is derepressed in Δ*hns*Δ*stpA* CFT073 (Fig. S3E). We conclude that all RfaH-regulated loci in CFT073 are occupied by H-NS–StpA, consistent with the RfaH elongation counter-silencing model.

10.1128/mbio.02662-22.3 FIG S3 New RfaH-bound ECs are initiation-silenced by H-NS in WT. (A) Average H-NS (red) and σ^70^ (orange and brown) ChIP/Input signal profiles for *wza* locus (Genome coordinate shown 2,425,770–2,531,025 adjusted to 0 for the translation start of the first gene in the locus) in WT and Δ*hns*Δ*stpA* strains. *ops* location indicated in magenta. ChIP signals were scaled locally (see Methods) for each IP to facilitate overlay. A new σ^70^ peak in Δ*hns*Δ*stpA* is highlighted with gray box. (B), (C), (D) Same as (A) but for *c1882* locus (Genome coordinates 1,715,065–1,718,565), *c4518* loci (Genome coordinates 4,304,935–4,303,105), *tssH* locus (Genome coordinates 3,221,545-3,238,195), respectively. (E) Model for newly identified RfaH-regulated loci illustrating silencing of promoters by H-NS in WT. In WT, H-NS–StpA (red, slate) binds promoters and occludes RNAP–σ^70^ from binding and initiating transcription. In Δ*hns*Δ*stpA*, RNAP initiation occurs either because an activator (green) becomes expressed, because H-NS no longer directly blocks promoters, or both. RNAP initiation occurs and RfaH is recruited to elongating RNAP at *ops*. Download FIG S3, TIF file, 1.5 MB. Copyright © 2022 Hustmyer et al. 2022 Hustmyer et al. https://creativecommons.org/licenses/by/4.0/ This content is distributed under the terms of the Creative Commons Attribution 4.0 International license.

### RfaH aids RNAP progression through H-NS-bound operons.

**FIG 3:**
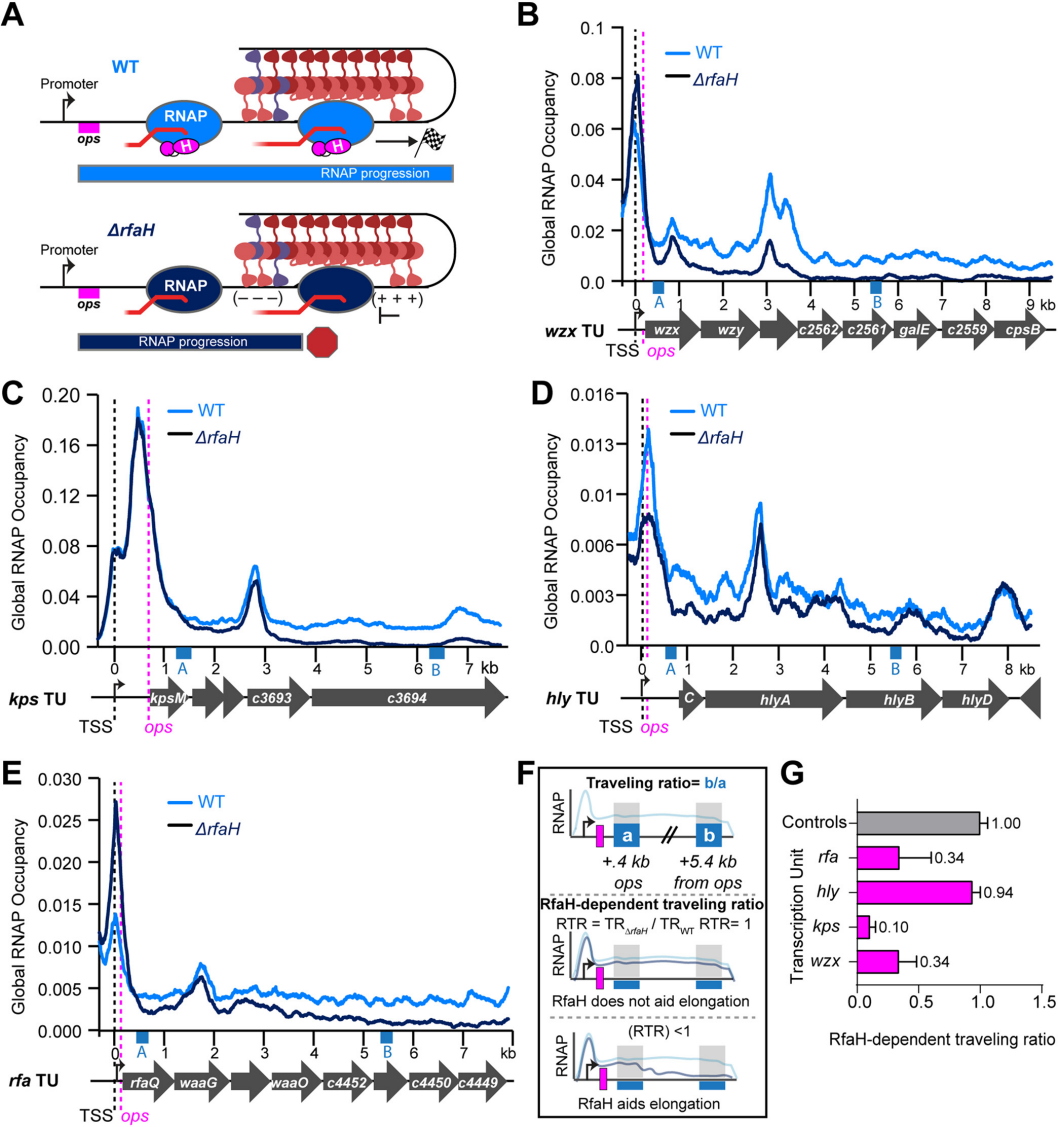
RfaH aids RNAP progression through H-NS filaments in CFT073. (A) Predicted effect of RfaH on RNAP progression. In WT CFT073, RfaH (magenta) binds RNAP (blue) at *ops* downstream of promoter (black arrow) and suppresses RNAP pausing promoted by topological stress that is increased by bridged H-NS–StpA, allowing RNAP to reach the end of the operon (checkered flag). In Δ*rfaH* CFT073, H-NS–StpA-induced pausing leads to ρ-dependent termination and failure of RNAP to produce full-length transcripts (red hexagon). (B) RNAP occupancy for WT (light blue) and Δ*rfaH* (dark blue) strains at the *wzx* TU (see Materials and Methods Text S1 for description of occupancy scaling). Pink dashed line, *ops.* Black dashed line, predicted TSS. RNAP occupancies are averages from three biological replicates. Blue boxes labeled *a* and *b* indicate windows used to calculate traveling ratio. (C), (D), (E) Same as (B) but for *kps*, *hly*, or *rfa* loci, respectively. (F) Traveling ratio (TR) calculation. RNAP ChIP signals in two 300-bp windows (blue boxes +0.4 kb from *ops* and + 5.4 kb from *ops* labeled *a* and *b*, respectively) were used to calculate traveling ratio as *b*/*a*. RfaH-dependent traveling ratio (RTR) was calculated by dividing the TR for Δ*rfaH* CFT073 by the TR for WT. (G) RTRs of RfaH-regulated TUs (magenta) compared to the average RTR for four control TUs not bound by RfaH (gray; average of *atp*, *dec*, *cyo*, and *his*; see Data set S3). Error was calculated as SD for three biological replicates.

**FIG 4:**
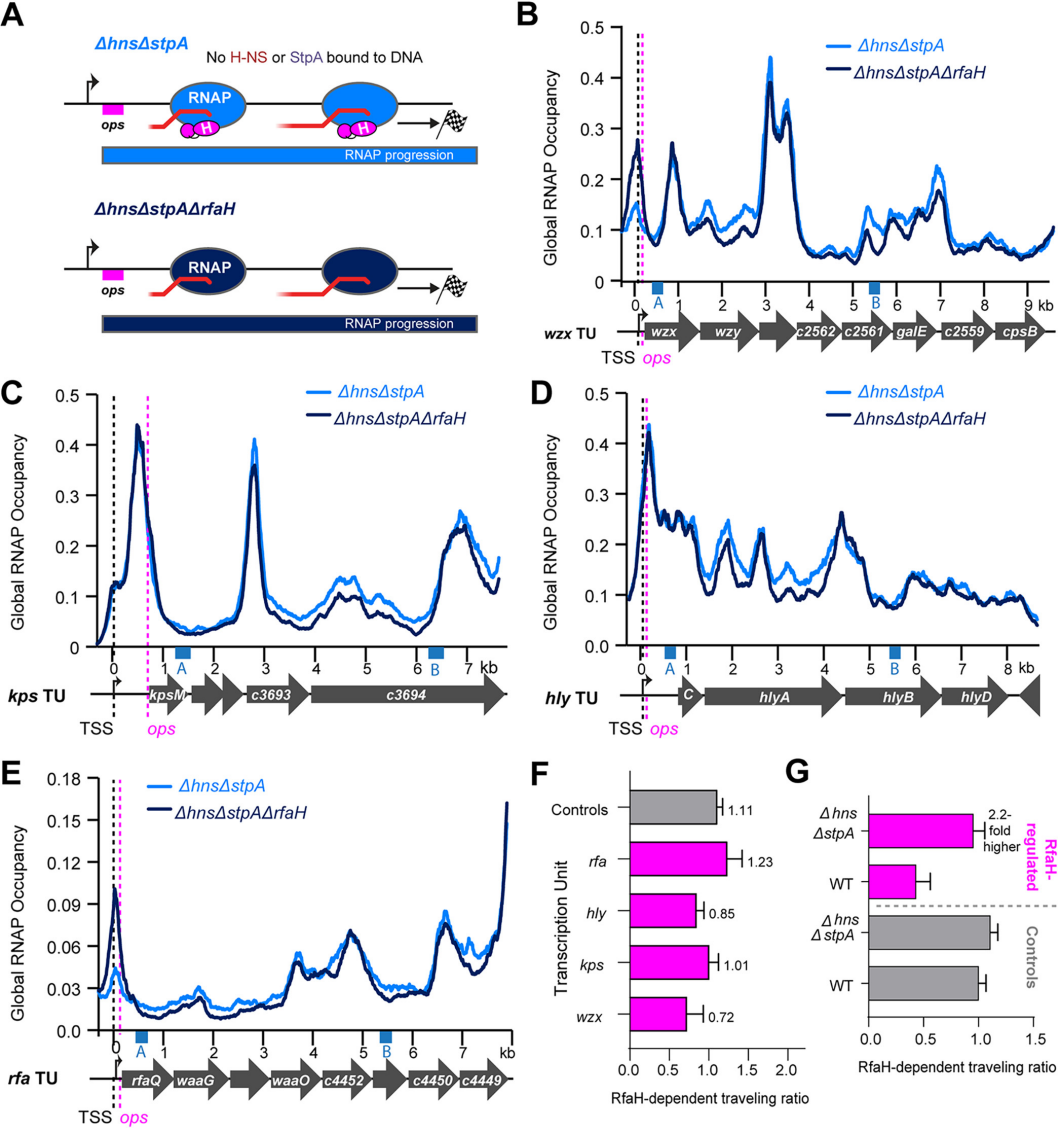
RNAP progression is independent of RfaH in the absence of H-NS and StpA. (A) Predicted lack of effect of RfaH on RNAP progression in Δ*hns*Δ*stpA* CFT073. RfaH (magenta) is still recruited to RNAP (blue) but lack of H-NS–StpA-stimulated pausing abrogates the counter-silencing effect of RfaH. (B) RNAP occupancy for *rfaH*^+^ (light blue) and Δ*rfaH* (dark blue) Δ*hns*Δ*stpA* CFT073 strains at the *wzx* TU as described in legend to Fig. 3. Occupancies represent averages of two biological replicates. (C), (D), (E) Same as (B) but for *kps*, *hly*, or *rfa* loci, respectively. (F) RTR for Δ*hns*Δ*stpA*Δ*rfaH* versus Δ*hns*Δ*stpA* CFT073 as described in legend to Fig. 3. Error is range from two biological replicates. (G) Comparison of average RTRs for RfaH-regulated TUs (magenta) versus control TUs (gray, same controls as in Fig. 3) for WT and Δ*hns*Δ*stpA* CFT073 strains.

To ask if RfaH aids RNAP elongation through H-NS–StpA filaments, we compared RNAP occupancy in the presence and absence of RfaH (Fig. 3A). For *wzx*, *kps*, *hly*, and *rfa*, RNAP occupancy decreased dramatically in Δ*rfaH* CFT073 to near background levels toward the ends of predicted TUs (Fig. 3B to E). To quantify RNAP progression through H-NS–StpA filaments, we calculated traveling ratios (TRs) for RNAP 5.4 kb downstream of *ops* relative to near *ops* (Fig. 3B to E, blue boxes, and Data set S3A). To estimate the RfaH effect, we calculated RfaH-dependent TRs (RTR = TR_Δ_*_rfaH_*/TR_WT_ [Fig. 3F]). RTRs were ≤0.1–0.34 for RfaH-regulated operons *wzx*, *kps*, and *rfa*, but ~1 for control operons not regulated by RfaH (Fig. 3G, and Fig. S4A and B). The higher RTR for *hly* (~0.9) may reflect the greater distance between *ops* and the translation start site at *hly* compared to other operons (~600 nt vs 40–67 for *wzx*, *kps*, and *rfa* [Data set S2A]). The long leader region appears to cause an immediate drop in RNAP levels when *rfaH* is deleted (Fig. 3D), resulting in an apparently high TR even though ρ and H-NS synergistically terminate transcription. The immediate drop in RNAP without RfaH was not observed in Δ*hns*Δ*stpA* CFT073 (Fig. 4D). RfaH levels at *hly* are the lowest of RfaH-bound WT genes (Fig. 2A and Fig. S2G), suggesting other TUs may be more affected by loss of *rfaH*. We conclude that RfaH aids RNAP elongation through H-NS filaments, consistent with the elongation counter-silencing model of RfaH action.

10.1128/mbio.02662-22.4 FIG S4 RfaH remains bound to ECs despite intragenic σ^70^ peaks. (A) RNAP occupancy for WT (light blue) and Δ*rfaH* (dark blue) strains at the *cyo* TU, which is not regulated by RfaH (Methods). Black dashed line, predicted TSS. RNAP occupancies are averages from 3 biological replicates. Blue boxes labeled *A* and *B* indicate windows used to calculate traveling ratio. (B) Summary of RTR calculated at control TUs (see extended Data set S3). (C) Average log-scaled RfaH-to-RNAP WT ChIP signal ratio (magenta) compared to average ChIP signals for σ^70^ (orange) and RNAP (blue) scaled to local maximal and minimal signals for each average (Methods). Averages are from 2 (RfaH) or 3 (σ^70^ and RNAP) replicates. Black arrows and dashed black line indicate predicted TSSs. Dashed magenta line indicates location of *ops* site. See also main Fig. 2. (D), (E), (F) same as (C) but for *kps*, *hly*, and *rfa* loci, respectively. Pink shaded box indicates area of interest described in text. Download FIG S4, TIF file, 1.3 MB. Copyright © 2022 Hustmyer et al. 2022 Hustmyer et al. https://creativecommons.org/licenses/by/4.0/ This content is distributed under the terms of the Creative Commons Attribution 4.0 International license.

10.1128/mbio.02662-22.10 Data set S3 (A) Traveling ratios for WT vs. *ΔrfaH* (B) Traveling ratios for *ΔhnsΔstpA* vs. *ΔhnsΔstpAΔrfaH* (C) Traveling ratios for *ΔstpA* vs. *ΔstpAΔrfaH*. Download Data Set S3, XLSX file, 0.02 MB. Copyright © 2022 Hustmyer et al. 2022 Hustmyer et al. https://creativecommons.org/licenses/by/4.0/ This content is distributed under the terms of the Creative Commons Attribution 4.0 International license.

### RfaH enhancement of RNAP elongation depends on the H-NS–StpA barrier.

We next asked if RfaH action depends on H-NS–StpA inhibition of RNAP elongation by comparing RNAP occupancy across RfaH-regulated TUs in Δ*hns*Δ*stpA* vs Δ*hns*Δ*stpA*Δ*rfaH* CFT073 (Fig. 4A). Without H-NS and StpA, RfaH had notably less effect on RNAP progression (Fig. 4B to E). RTRs (TR_Δ_*_hns_*_Δ_*_stpA_*_Δ_*_rfaH_*/TR_Δ_*_hns_*_Δ_*_stpA_*) averaged ~0.95 compared to ~1.1 at control TUs (Fig. 4F). Collectively, RTRs were 2.2-fold higher than when H-NS and StpA were present (compare Fig. 4F to 3G, Fig. 4G, and Data set S3B). These results indicate that RfaH enhancement of RNAP elongation depends strongly on the presence of H-NS–StpA filaments that impede RNAP elongation, consistent with the elongation counter-silencing model of RfaH action.

### StpA enhances RfaH-mediated regulation of RNAP progression.

**FIG 5:**
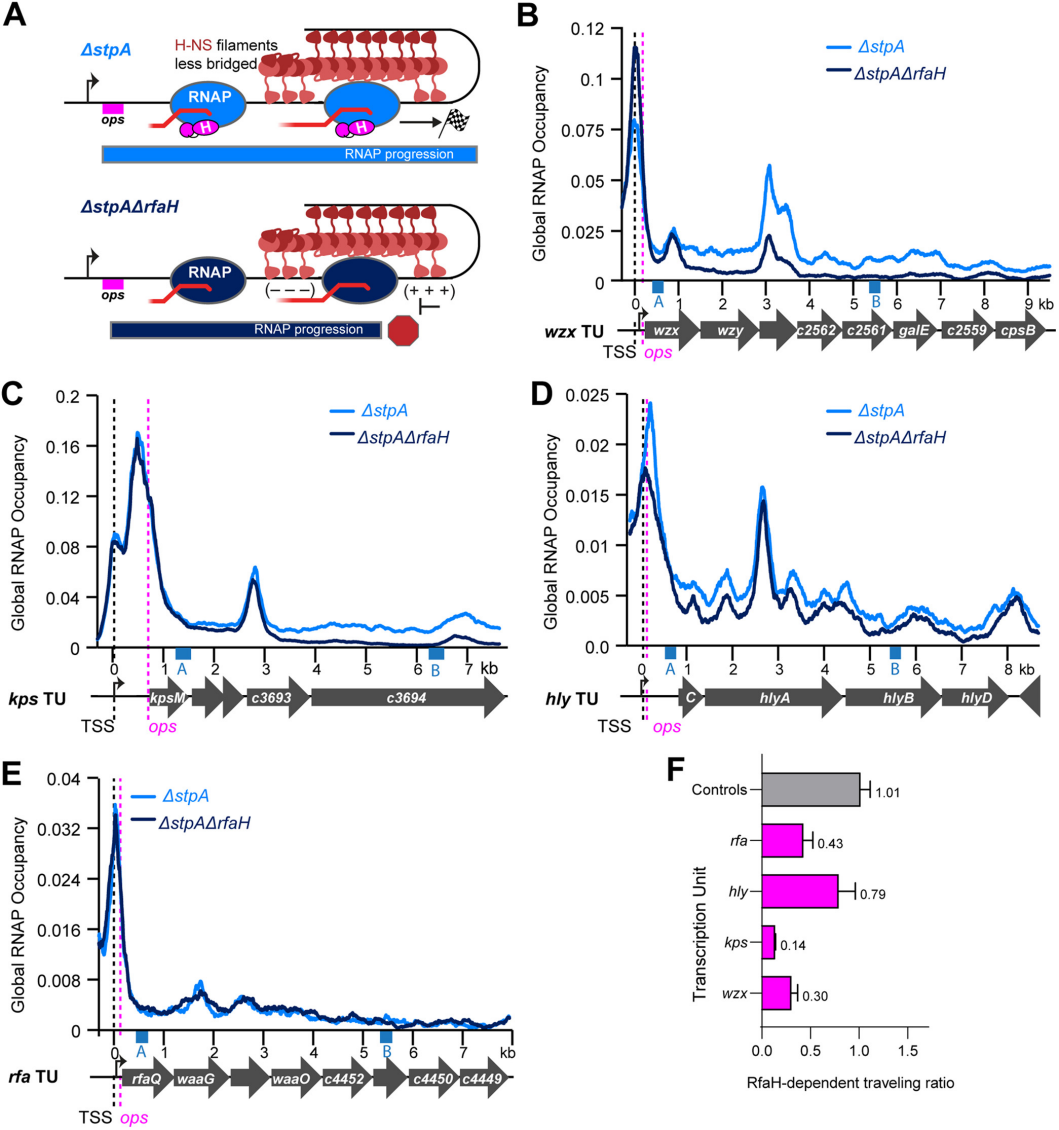
Deletion of bridging enhancer StpA modestly aids RNAP elongation without RfaH. (A) Predicted effect of RfaH on RNAP progression in Δ*stpA* and Δ*stpA*Δ*rfaH* CFT073. In Δ*stpA*, H-NS filaments (red) will be less bridged, increasing RNAP progression. RNAP progression will decrease in the absence of RfaH, but the magnitude of effect may be less than for WT CFT073. (B) RNAP occupancy for Δ*stpA* (light blue) and Δ*stpA*Δ*rfaH* (dark blue) CFT073 strains at the *wzx* TU as described in legend to Fig. 3. Occupancies represent averages of three biological replicates. (C), (D), (E) Same as (B) but for *kps*, *hly*, or *rfa* loci, respectively. (F) RTR for Δ*stpA*Δ*rfaH* versus Δ*stpA* CFT073 as described in legend to Fig. 3. Error was calculated as SD from three biological replicates.

**FIG 6:**
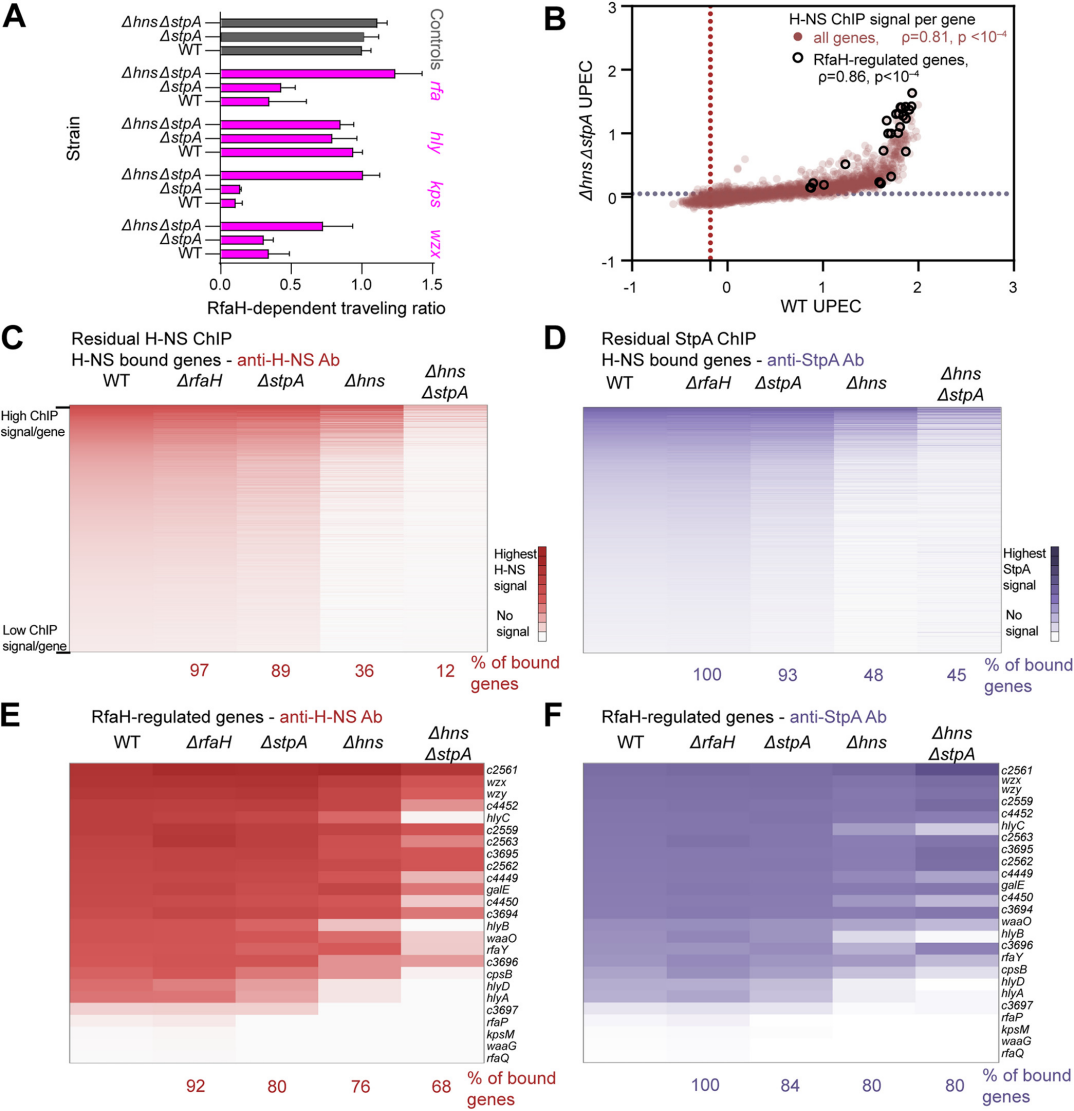
H-NS and StpA exhibit high affinity for RfaH-regulated genes. (A) RTR for all CFT073 strains tested compared (same as Fig. 3, Fig. 4, and 5). (B) Scatterplots of the average H-NS ChIP/Input signals for all genes in Δ*hns*Δ*stpA* versus WT CFT073. RfaH-regulated genes are outlined in black. Data are averages from three biological replicates. Spearman correlation parameters are given for all genes (red) and for RfaH-regulated genes (black). (C) Heat map of average H-NS ChIP signals per gene for WT, Δ*rfaH*, Δ*stpA*, Δ*hns*, Δ*hns*Δ*stpA.* Average ChIP values were scaled 0–1 for each strain (Data set 1E) and sorted from 1 (top) to 0 (bottom, lowest value scored as H-NS–bound) for WT CFT073. (D) Heat map of average StpA ChIP signals per gene for WT, Δ*rfaH*, Δ*stpA*, Δ*hns*, Δ*hns*Δ*stpA* scaled and sorted as described for (C). (E) Same as (B) but for only RfaH-regulated genes. (F) Same as (D) but for only RfaH-regulated genes.

StpA increases H-NS filament bridging, which slows RNAP topologically (16, 23). Therefore, we next asked if eliminating StpA alone would reduce RfaH enhancement of RNAP progression (Fig. 5A). The RTR (TR_Δ_*_stpA_*_Δ_*_rfaH_*/TR_Δ_*_stpA_*) was similar to the RTR in WT CFT073 for *wzx* and *hly* but was modestly increased for *rfa* and *kps* (compare Fig. 5F and 3G [Data set S3C and Fig. 6A]). These results indicated that both H-NS and StpA impede RNAP progression, but do not fully explain the effect of H-NS–StpA filaments on RfaH regulation. Of note, the bridging enhancer Hha (19, 23), its multiple paralogs (21, 38), and H-NS paralog Hfp (33, 38) remain in Δ*stpA* CFT073. We conclude the modest but detectable effects of removing StpA are consistent with the elongation counter-silencing model of RfaH action.

### Intragenic RNAP–σ^70^ binding does not contribute to RfaH-mediated elongation counter-silencing.

Many horizontally acquired and H-NS-silenced genes contain promoter-like sequences that bind RNAP–σ^70^ and can initiate intragenic, noncoding transcription (2, 15, 62, 63). Consistent with these observations, the RfaH-regulated TUs in CFT073 exhibited some intragenic σ^70^ ChIP-seq peaks (Fig. 2B to E). To ask if transcription from these intragenic promoters contributed to RfaH-mediated counter-silencing, we examined RNAP, RfaH, and RfaH/RNAP ChIP signals around the σ^70^ peaks (Fig. S4C to F, pink boxes). The apparent RNAP and RfaH levels and the RfaH/RNAP ratio were constant on either side of these intragenic σ^70^ peaks, suggesting that transcription from these sites did not contribute to RfaH-mediated transcription through H-NS filaments. σ^70^ ChIP-seq signal was also similar at RfaH-regulated TUs in the presence and absence of RfaH (Fig. S5A and Fig. S5C to F).

10.1128/mbio.02662-22.5 FIG S5 Intragenic RNAP–σ^70^ binding does not contribute to RfaH-mediated elongation counter-silencing. (A) Scatter plot of the average σ^70^ ChIP/Input signal (log_10_ scaled) for all genes in Δ*rfaH* vs. WT CFT073. Spearman correlation coefficients are reported for σ^70^ on all genes (orange) and on RfaH-regulated genes (black). Data are averages of 3 biological replicates. (B) Same as (A), but for σ^70^ in Δ*hns*Δ*stpA* vs. WT CFT073. Signals are averages of at least 2 biological replicates. (C), (D), (E), (F) RNAP (blue) and σ^70^ (orange) ChIP signal profiles for WT (light colors) and Δ*rfaH* (dark colors) CFT073 strains at *wzx*, *kps*, *hly*, and *rfa*, respectively. ChIP signals are scaled to global or local maxima and minima for RNAP and σ^70^, respectively (see legends to Fig. 2 and 3, and Methods). Blue boxes labeled *A* and *B* indicate windows used to calculate traveling ratio. Data are averages of three biological replicates. (G) Average log-scaled RfaH-to-RNAP WT ChIP signal ratio (magenta) compared to average ChIP signals for σ^70^ (orange) and RNAP (blue) in Δ*hns*Δ*stpA* scaled to local maximal and minimal signals for each average (Methods). Averages are from two (RfaH, RNAP) or three (σ^70^) replicates. Black arrows and dashed black line indicate predicted TSSs. Dashed magenta line indicates location of *ops* site. (H), (I), (J) same as (G) but for *kps*, *hly*, and *rfa* loci, respectively. Download FIG S5, TIF file, 2.4 MB. Copyright © 2022 Hustmyer et al. 2022 Hustmyer et al. https://creativecommons.org/licenses/by/4.0/ This content is distributed under the terms of the Creative Commons Attribution 4.0 International license.

As expected, we observed new intragenic σ^70^ ChIP-seq signal when H-NS and StpA were absent (Fig. S5B). However, we observed no major changes in RfaH/RNAP ratios downstream of σ^70^ ChIP-seq peaks in Δ*hns*Δ*stpA* strains (Fig. S5G to J). Thus, greater processivity of RNAP, rather than increased transcription from intragenic promoters, appears to explain the increase in TR in Δ*hns*Δ*stpA* versus WT strains (Fig. 6A). We conclude that RfaH is primarily responsible for counter-silencing H-NS within RfaH-regulated TUs.

### RfaH-enhanced transcript elongation does not displace H-NS–StpA filaments.

We next asked if RfaH-enhanced RNAP elongation by displacing or transiently remodeling H-NS–StpA filaments by determining H-NS ChIP signal on genes in WT vs Δ*rfaH* CFT073 (Fig. S6A). H-NS remained bound to all RfaH-regulated loci in the presence of RfaH, indicating that RNAP elongation occurs without extensive removal of H-NS–StpA filaments from DNA (Fig. S6B). Notably, H-NS–StpA filaments at RfaH-regulated loci gave among the highest H-NS and StpA ChIP signals (Fig. S6B and Data set S1E), suggesting these loci may contain multiple high affinity binding sites for H-NS and its paralogs. We conclude RfaH must remodel the filament by helping RNAP transiently displace H-NS and allow transcription. Transient H-NS displacement would not be expected to alter H-NS ChIP signal averaged across large numbers of cells (75). Continued association of H-NS–StpA with DNA during RfaH-regulated transcription is consistent with the topological model of bridged H-NS–StpA inhibition of transcript elongation, with transient displacement of DNA-binding domains to allow RNAP progression (17, 23).

10.1128/mbio.02662-22.6 FIG S6 H-NS exhibits high affinity for RfaH-regulated transcription units. (A) Heat maps of H-NS ChIP signal in 1.4 kb tiling windows across the genome of WT (upper) and Δ*rfaH* (lower) CFT073 strains (see legend to Fig. S1 and Methods for description of scaling Text S1). (B) Scatter plot of average H-NS ChIP signal (log_10_ scaled) for all genes in WT vs. Δ*rfaH* CFT073 strains. Black dots, RfaH-regulated genes. (C) Quantitation of H-NS (red), StpA (purple) and Hfp in CFT073 strains from western blots (bar chart; see Methods Text S1). Hfp detected in cell extracts with anti-StpA antibodies migrates slightly faster than H-NS (top panel). This blot image is cropped, and contrast uniformly adjusted from a section of Fig. S1G. Using known amounts of *in vitro* synthesized, Flag-His-tagged-Hfp, -H-NS, or -StpA as internal standards added to cell lysates (right panel) yielded estimates of ~40,000 H-NS monomers/cell in WT and ~6,000 Hfp monomers/cell in Δ*hns*Δ*stpA* CFT073. Images in right panel are from 2 separate blots that are uniformly image-enhanced and contain (WT) 0.6 μg cell lysate and 2.6 ng tagged H-NS or (Δ*hns*Δ*stpA*) 2 μg cell lysate and 3.9 ng tagged-Hfp. Anti-StpA probing likely overestimates StpA copy number due to cross-reactivity with H-NS and Hfp (indicated by +* on bar chart). Standard deviations from 3 biological replicates are reported. (D) Top: Representative H-NS occupancy normalized H-NS IP/Input in WT (light brown) and *ΔhnsΔstpA* (red). Asterisks represent WT RfaH-regulated loci. Bottom: Heat maps of H-NS (red) and StpA (purple) occupancy signal in 1.4 kb tiling windows across the genome for WT, Δ*rfaH*, Δ*stpA*, Δ*hns*, and Δ*hns*Δ*stpA* CFT073 strains. (E) Fractions of RfaH-regulated genes bound by H-NS paralogs in WT, *ΔstpA*, *Δhns*, and *ΔhnsΔstpA* CFT073 strains (Data set S1E). (F) Fractions of non-RfaH-regulated Class IV genes (high RNAP and H-NS occupancy; see Fig. 1E) that are bound by H-NS paralogs (i.e., score as bound by H-NS ChIP-seq) in WT, *ΔstpA*, *Δhns*, and *ΔhnsΔstpA* CFT073 strains (Data set S1E). (G) Enrichment of features associated with H-NS binding in the set of genes bound using the anti-H-NS antibody (see Methods Text S1 and Shen et al., 2022 *iScience* 25:104429). Lines on each distribution represent the median. Download FIG S6, TIF file, 2.8 MB. Copyright © 2022 Hustmyer et al. 2022 Hustmyer et al. https://creativecommons.org/licenses/by/4.0/ This content is distributed under the terms of the Creative Commons Attribution 4.0 International license.

### RfaH-regulated genes exhibit highest affinity for H-NS paralogs.

Even in strains lacking both H-NS and StpA, RfaH affected RNAP progression (Fig. 6A). ChIP-seq using anti-H-NS or anti-StpA antibodies gave residual signals on a few loci in *ΔhnsΔstpA* strains, including RfaH-regulated genes (Fig. 6B and Fig. S6D). We hypothesized this signal was generated by the minor H-NS paralog in CFT073, Hfp. The residually bound loci might reflect sites with highest affinity for H-NS and its paralogs, since high affinity sites would preferentially recruit paralogs present at lower concentrations. Residual Hfp binding could explain the residual RfaH counter-silencing effect.

To test these hypotheses, we examined anti-H-NS and anti-StpA ChIP–signal distributions in strains with deletions in *hns*, *stpA*, or both (Data set S1E). These signals were similarly distributed in an *hns* deletion strain but to ~70% of the genes detected in WT CFT073 (Fig. 6C and D, and Data set S1F). StpA levels in Δ*hns* may be lower than in WT because StpA not bound to H-NS is degraded by Lon protease (76). When both H-NS and StpA were deleted, only 12% of genes remained bound based on anti-H-NS ChIP-seq. We confirmed that this residual signal represents, at least in large part, H-NS and StpA antisera targeting Hfp in CFT073 *ΔhnsΔstpA* using quantitative Western blots of cell lysates and *in vitro* synthesized, tagged-Hfp. In *ΔhnsΔstpA*, Hfp levels are ~15% of the H-NS level in WT (~6,000 Hfp/cell versus ~40,000 H-NS/cell [Fig. S6C]).

Many of the Hfp-bound genes were RfaH-regulated (Fig. 6C and D). Overall, ~68% of RfaH-regulated genes retained signal (Fig. 6E and F, and Fig. S6E) compared to ~5% of genes with strong RNAP and H-NS signals in WT CFT073 (class IV [Fig. S6F]). These results suggest that RfaH-regulated genes contain stronger H-NS binding sites than non-RfaH- regulated class IV genes.

To test this, we scored CFT073 genes for DNA sequence and shape features associated with high affinity H-NS binding (77). High A/T content, TA steps, narrow minor-groove width, and lower electrostatic potential favor H-NS binding, and all were enriched in CFT073 genes bound by H-NS–StpA (Fig. S6G). Strikingly, these features were further enriched in the sets of genes retaining ChIP signal in Δ*stpA*, Δ*hns*, and Δ*hns*Δ*stpA* strains. This result strongly suggests that genes retaining ChIP signal at lower levels of H-NS and its paralogs have higher affinity for these proteins. Thus, high affinity for H-NS and its paralogs is a characteristic of RfaH-regulated genes, suggesting that strong silencing of transcript elongation could be a component of the elongation counter-silencing mechanism.

## DISCUSSION

**FIG 7:**
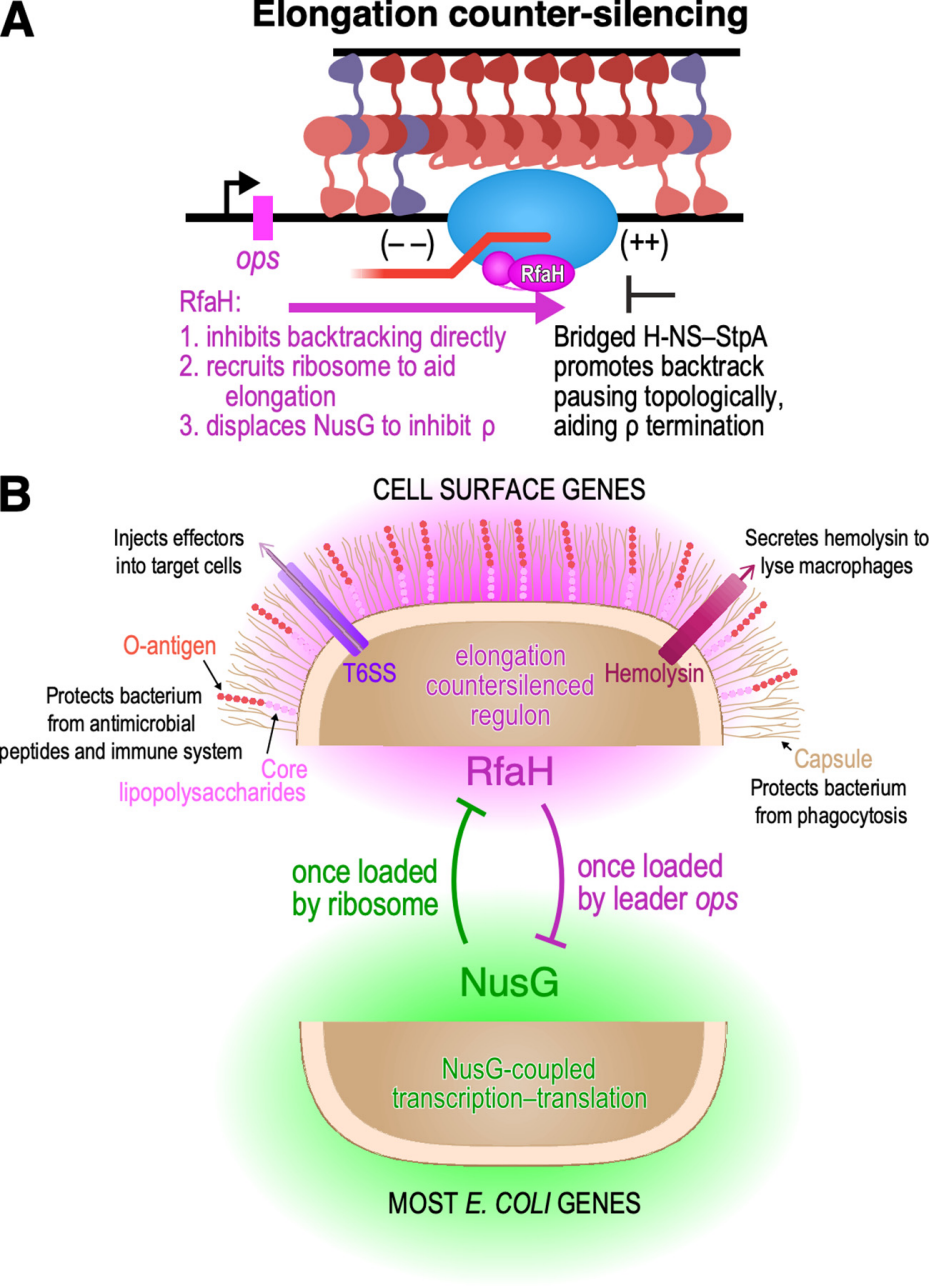
RfaH is an elongation counter-silencer of H-NS–StpA gene silencing. (A) The elongation counter-silencing model. RfaH (magenta) acts as an elongation counter-silencer by binding to RNAP at a subset of H-NS–bound loci that contain an *ops* site. Bridged H-NS and StpA promote backtrack pauses topologically, enabling ρ-dependent termination. RfaH counter-silences H-NS–StpA gene silencing by directly inhibiting backtracking of RNAP, by recruiting a ribosome that can aid RNAP elongation, and by displacing NusG, which prevents NusG-stimulation of ρ-dependent termination. (B) The RfaH regulon in CFT073 based on loci bound by RfaH compared to the untargeted action of NusG (green). The RfaH targets in CFT073 all encode cell envelope components including enzymes for synthesizing cell envelop components, lipopolysaccharides and *O*-antigen, a type-6 secretion system (T6SS), hemolysin, and capsule polysaccharides.

We report the genomic distributions of H-NS, StpA, RfaH, σ^70^, and RNAP in six derivatives of the pathogenic E. coli strain CFT073, resulting in four new insights: (i) H-NS and StpA are distributed indistinguishably on ~18% of the CFT073 genome; (ii) all RfaH-regulated operons are bound by H-NS–StpA; (iii) RfaH aids RNAP elongation through H-NS–StpA filaments, acting as an elongation counter-silencer of H-NS–StpA gene silencing (Fig. 7A), and (iv) StpA aids H-NS hindrance of RNAP elongation, consistent with a role of bridging in effects of H-NS on transcript elongation *in vivo.*

### RfaH exclusively targets H-NS–StpA-bound, pathogenicity-related operons via 5′-leader *ops* sites.

We found that the RfaH regulon consists of eight operons in CFT073 (Fig. 7B), with *ops* in their 5′ leader regions (Data set S2A). RfaH does not bind *ops*-like sites within 22 coding regions of other operons, suggesting that NusG-mediated transcription–translation coupling, once established, blocks RfaH binding. The RfaH regulon includes multiple pathogenicity functions (hemolysin production, cell wall and *O-*antigen synthesis, capsule formation, uncharacterized CFT073 specific proteins, and apparent regulators of type VI secretion) (Fig. 7B), likely explaining why loss of RfaH attenuates colonization of UPEC in mouse models (54).

All eight RfaH-bound loci were also bound by gene-silencing H-NS–StpA nucleoprotein filaments. CFT073 encodes 4818 coding genes, 18% of which are bound by H-NS–StpA, making the odds of all 25 RfaH-regulated genes being bound by H-NS–StpA remote (<10^−19^ versus a purely random assortment). These loci may have among the highest affinity sequences for H-NS–StpA in the CFT073 genome and preferentially bind low levels of the H-NS paralog Hfp (33, 38) present in Δ*hns*Δ*stpA* CFT073 (Fig. 6E and F, and Fig. S6G). The bridging properties of Hfp are uncharacterized, but Hfp can bind DNA and compensate for H-NS loss by silencing *bgl* and RfaH-regulated *kps* (33). High affinity for H-NS and its paralogs may poise the RfaH regulon for effective counter-silencing.

### RfaH is a counter-silencer of H-NS–StpA inhibition of transcript elongation.

The evolutionary origins of RfaH have been speculated (43, 78), but no mechanistic hypothesis has been considered. RfaH may have arisen in response to selective pressure to express horizontally transferred operons that are silenced by H-NS and its paralogs. H-NS targets A/T-rich genes horizontally transferred into enteric bacteria (7–9, 58). Silencing requires inhibiting transcription initiation at the acquired promoters (2, 15, 62, 63), and also inhibiting elongation into new DNA because insertion of horizontally transferred DNA often occurs downstream of highly active promoters (79). Thus, H-NS bridging may have evolved in part to inhibit transcript elongation by topologically stimulating pausing and ρ-dependent termination (12, 17, 23). Expression of some horizontally transferred genes may be beneficial to bacteria, for example by enabling cell surface alterations to evade host immunity. By deriving from NusG an operon-specific regulator requiring only a short *ops* sequence for recruitment (i.e., RfaH), enterobacteria may have evolved a regulatory mechanism enabling expression of useful horizontally transferred genes ordinarily silenced by H-NS.

Our findings directly demonstrate that RfaH is an elongation counter-silencer of H-NS–StpA filaments *in vivo*. First, RfaH is exclusively recruited to H-NS–StpA-bound loci (Fig. 2A). Second, deleting RfaH severely impedes RNAP progression through H-NS–StpA filaments (Fig. 3). Third, RfaH requirement for efficient RNAP progression is lessened in the absence of H-NS, StpA, or both (Fig. 6A).

We posit that RfaH counter-silences H-NS–StpA filaments through multiple direct and indirect routes (Fig. 7A). First, RfaH directly prevents back-tracked pausing that H-NS–StpA filaments promote (17, 50). The NTD of RfaH can suppress backtracked pauses stimulated by bridged H-NS *in vitro* (17). *In vivo*, H-NS–StpA filaments remain robustly bound to DNA in the absence of RfaH (Fig. S6A and B); RNAP progression is severely impeded (Fig. 3 and Fig. 6A) because H-NS–StpA topologically traps RNAP to stimulate termination. By suppressing backtracked pausing, which bridged H-NS–StpA filaments stimulate, RfaH helps RNAP elongate through topologically-constrained domains created by bridged H-NS–StpA filaments. Second, RfaH likely recruits the ribosome (45), which stabilizes RfaH association and promotes RNAP elongation by preventing back-tracked pausing (69, 70); coupled ribosome–RfaH elongation may also help RNAP elongate through H-NS–StpA filaments. Third, RfaH, which does not bind ρ, will counter-silence H-NS–StpA filaments by excluding NusG and thus inhibiting ρ-dependent termination (49, 67).

### RfaH counter-silencing likely depends on H-NS–StpA bridging.

Bridged, but not linear, H-NS–StpA stimulates transcriptional pausing. Pausing slows RNAP and enables ρ-dependent termination by exacerbating torsional strain generated by transcript elongation (17, 23). The topologically closed DNA domains created by bridging prevent (+) supercoiling generated in front and (–) supercoiling generated behind RNAP from being relieved by DNA rotation and possibly by topoisomerases whose access could be restricted by H-NS–StpA. The consequent increase in supercoiling upon transcript elongation disfavors forward translocation and favors backtrack pausing by RNAP (Fig. 7A).

StpA and Hha both favor bridging relative to H-NS alone (19, 23). H-NS can sequester one DBD per dimer in hemi-sequestered, linear filaments, whereas StpA appears unable to sequester its DBD and Hha blocks the sequestration site (19, 20, 23). Thus, StpA and H-NS–Hha filaments are constitutively bridged and inhibit transcript elongation much more strongly than H-NS–only filaments (23). Our finding that deletion of StpA decreases the impact of RfaH on expression of some RfaH-regulated operons in CFT073 is fully consistent with this topological model of H-NS inhibition of transcript elongation. Because StpA is distributed randomly with H-NS among A/T-rich CFT073 genes (Fig. 1D), removal of StpA should modestly relieve the effect of RfaH on transcript elongation. Quantitative analysis of RNAP occupancy revealed this predicted modest effect in Δ*stpA* CFT073 (Fig. 5). We were unable to test contributions of Hha to RfaH regulation in CFT073 because Δ*hha* CFT073 strains were unstable and because CFT073 encodes multiple Hha paralogs. A recent study of E. coli K-12 provides indirect evidence implicating Hha in RfaH-mediated regulation that is consistent with our counter-silencing model of RfaH regulation (78).

Bridging by H-NS is favored at low ratios of H-NS to available DNA-binding sites (17). RfaH-regulated genes exhibit apparent higher affinity for reduced levels of H-NS paralogs (Fig. 6 and Fig. S6), consistent with enhanced bridging at these loci. With high affinity for H-NS DNA-binding domains, loci could more effectively compete with sequestration sites on filaments bound to a distinct DNA segment, facilitating bridging. Indeed, among 108 H-NS-bound, non-RfaH-regulated genes that exhibit high levels of elongating RNAP (class IV genes [Fig. 1E]), over 80% lost occupancy by H-NS paralogs in a Δ*hns* strain and ~95% lost occupancy in a Δ*hns*Δ*stpA* strain (Fig. S6F), suggesting weaker affinity for H-NS paralogs and contrasting with 76% and 68% retention of occupancy of 25 RfaH-regulated genes, respectively, in the same strains (Fig. 6E and Fig. S6E). These differences suggest class IV genes may be less susceptible to H-NS bridging and potentially explain why transcript elongation is possible on these genes without RfaH. Conversely, RfaH-regulated genes may have a greater propensity for pause-enhancing bridging that increases the dynamic range of the elongation counter-silencing mechanism.

Overall, our findings establish that RfaH regulates at least 25 genes in WT pathogenic E. coli by an elongation counter-silencing mechanism. Elongation counter-silencing is likely to be an important regulatory feature in other proteobacteria that utilize H-NS paralogs and encode RfaH (80). RfaH may counter-silence H-NS filaments to enable pathogenesis by upregulating virulence genes, making it a promising drug target. Given the wide distribution of NusG paralogs in bacteria (43) but relatively narrow distribution of H-NS paralogs (21), it will be interesting to learn if other bacterial chromatin proteins can be elongation counter-silenced in diverse bacterial lineages.

## MATERIALS AND METHODS

### Strain construction.

UPEC strain CFT073 (81) or E. coli K-12 strain RL3000, a prototrophic MG1655 derivative (82), were used for all experiments. A recombination protocol using E. coli CFT073 harboring λ*red* recombination machinery was used to replace genes in-frame with kanamycin-resistance cassettes as described previously (83). CFT073-specific phage ΦEB49 was used to transduce deletions into WT CFT073 (84). Strain construction is described in detail in extended methods (Text S1).

10.1128/mbio.02662-22.7 Text S1 Extended details on methodology used in Hustmyer *et al.* Download Text S1, PDF file, 0.3 MB. Copyright © 2022 Hustmyer et al. 2022 Hustmyer et al. https://creativecommons.org/licenses/by/4.0/ This content is distributed under the terms of the Creative Commons Attribution 4.0 International license.

### Chromatin-immunoprecipitation sequencing.

ChIP-seq was performed as previously described (85) with modifications described in extended methods (Text S1). Strains were grown in MOPS rich defined media (RDM) supplemented with 0.2% glucose (86) (TekNova) and cross-linked at an apparent OD_600_ ~0.4 with 1% formaldehyde. H-NS, StpA, RfaH, Beta, and σ^70^ IPs, and no antibody and input controls were performed as described (61, 85) with modifications described in extended methods (Text S1). DNA sequencing paired-end libraries were prepared using NEBNext Ultra II DNA library reagents according to the manufacturer’s protocols and sequenced on Illumina NovaSeq 6000 or MiSeq sequencers.

### ChIP-seq analysis.

CFT073 reads were aligned to updated RefSeq annotations detailed in GenBank accession number NC_004431.1. The genome was divided into 5-bp bins and read coverage per bin was calculated and scaled by the median coverage of all bins for each IP, as described in (87). Data were then normalized to coverage from an input control using the ratio of median-scaled read coverage divided by the input median-scaled read coverage, unless otherwise noted. Analysis scripts are available at https://github.com/cmhustmyer/2022_hustmyer. Details of analysis and occupancy normalization are described in the extended methods (Text S1).

### Data availability.

ChIP-seq data sets were deposited at Gene Expression Omnibus (GEO) with accession code GSE212064.
