# Supplementary figures and images for: RfaH Counter-Silences Inhibition of Transcript Elongation by H-NS–StpA Nucleoprotein Filaments in Pathogenic Escherichia coli
Source: mBio. 2022 Oct 20;13(6):e02662-22. doi: 10.1128/mbio.02662-22 (PMC9765446; doi:10.1128/mbio.02662-22)

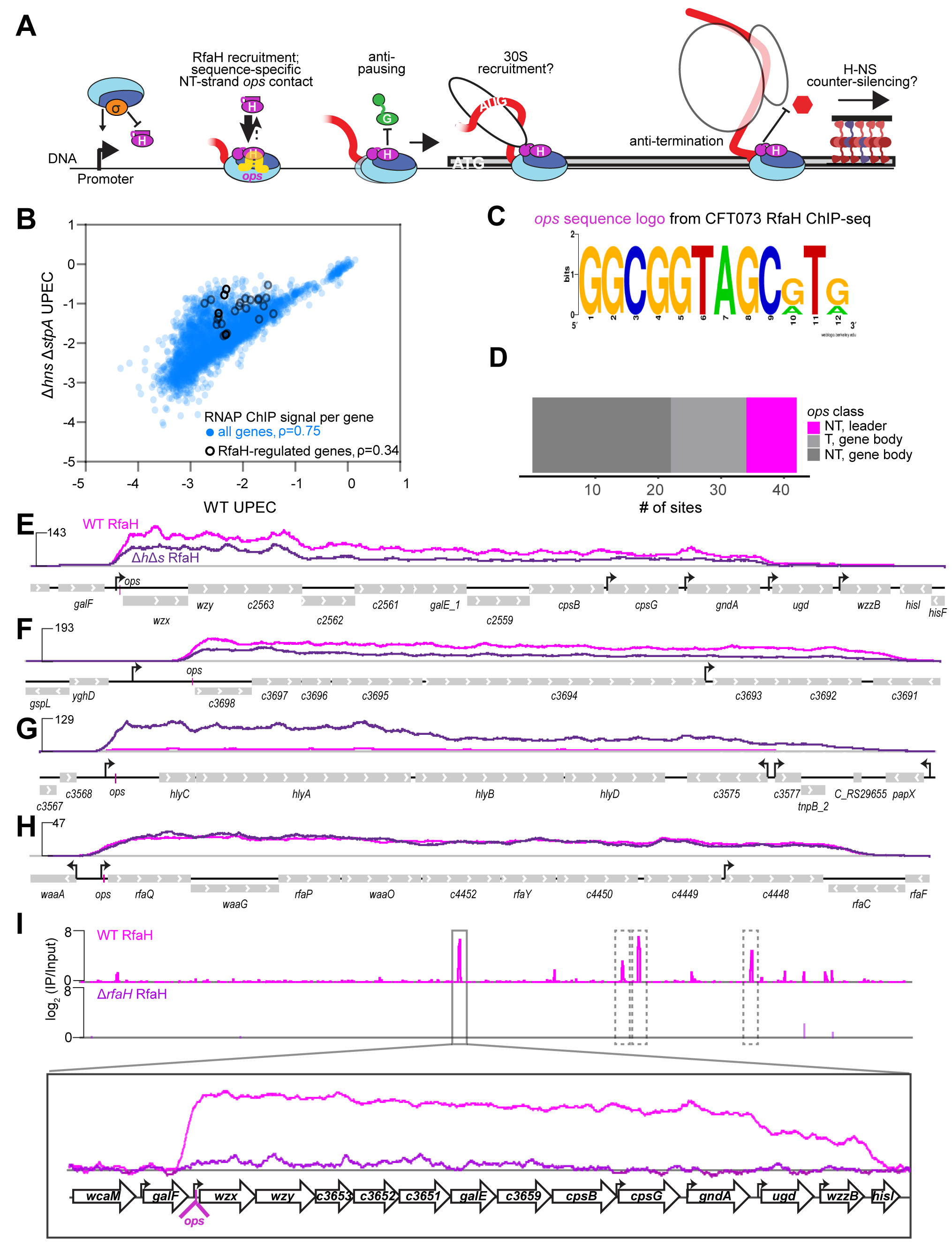

Supplement: FIG S2 [file mbio.02662-22-s0002.tif]

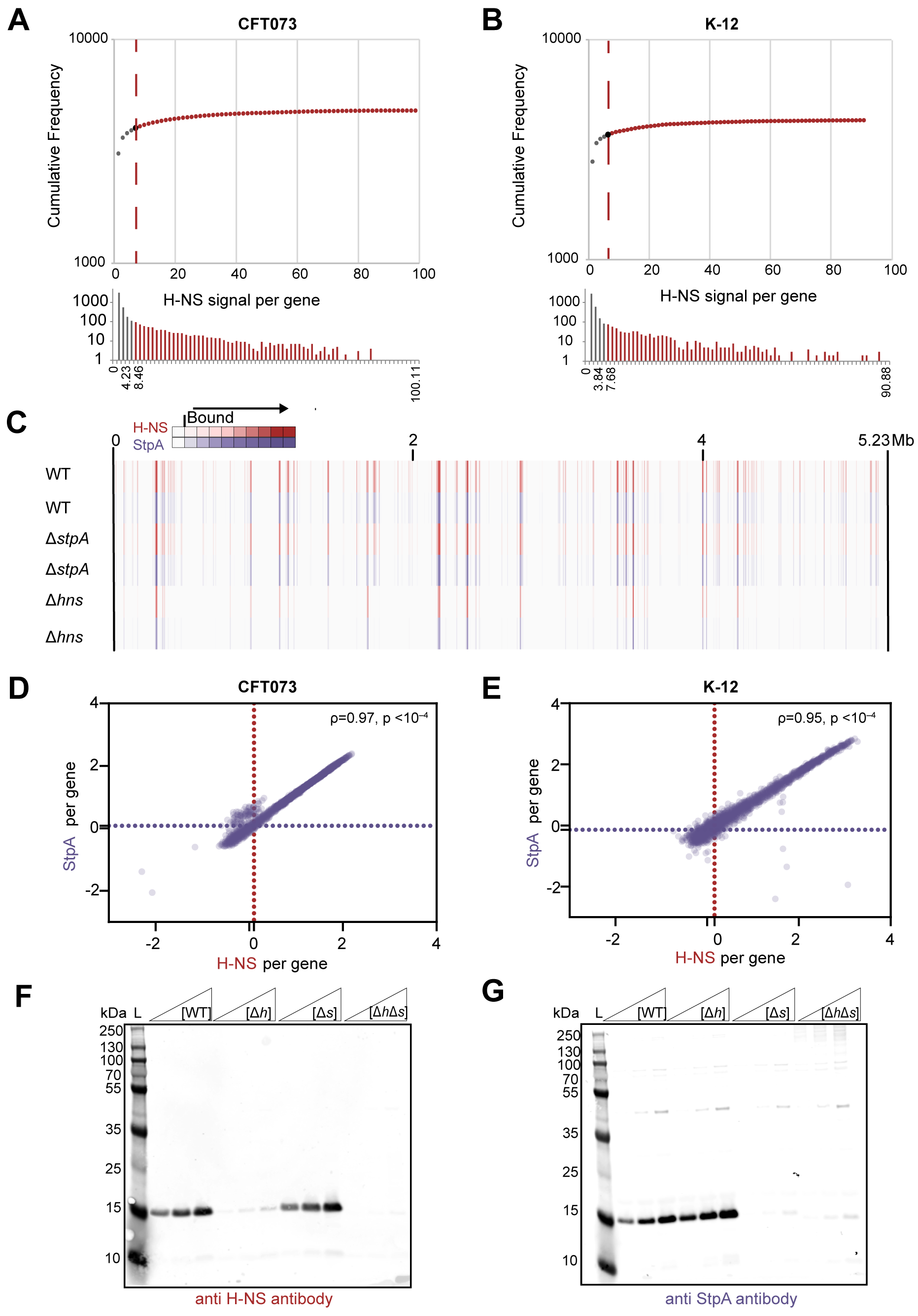

Supplement: FIG S1 [file mbio.02662-22-s0001.tif]

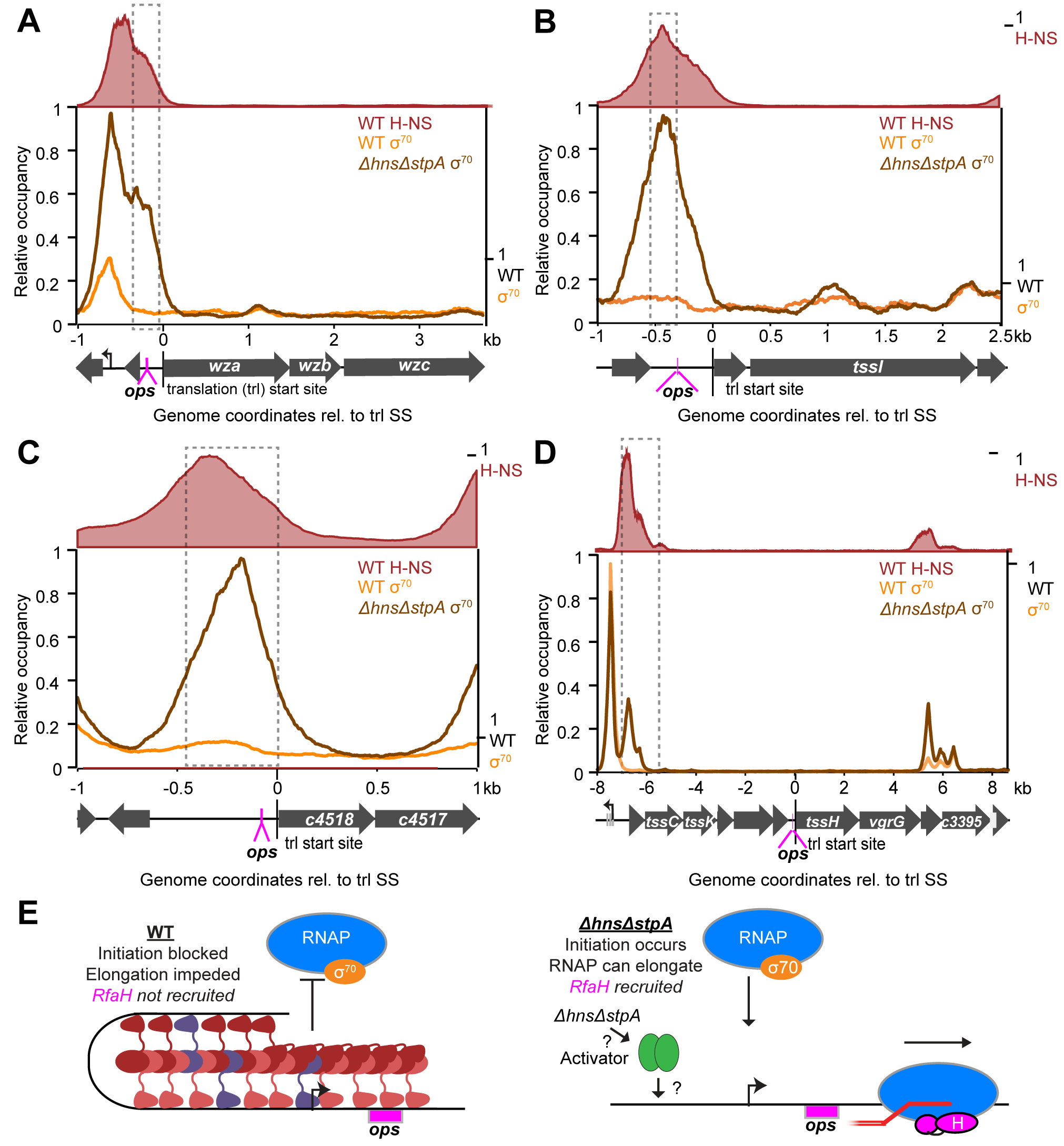

Supplement: FIG S3 [file mbio.02662-22-s0003.tif]

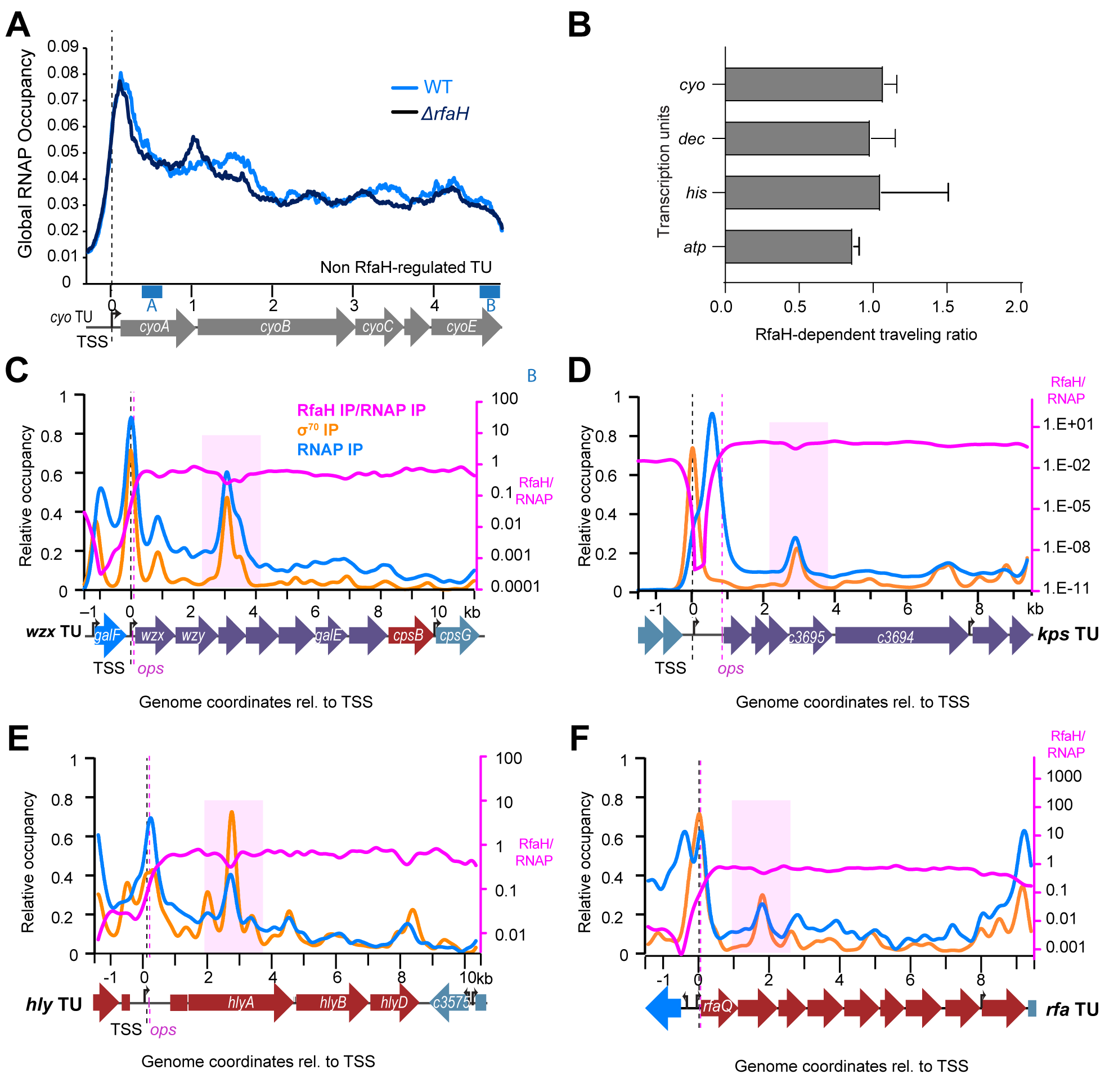

Supplement: FIG S4 [file mbio.02662-22-s0004.tif]

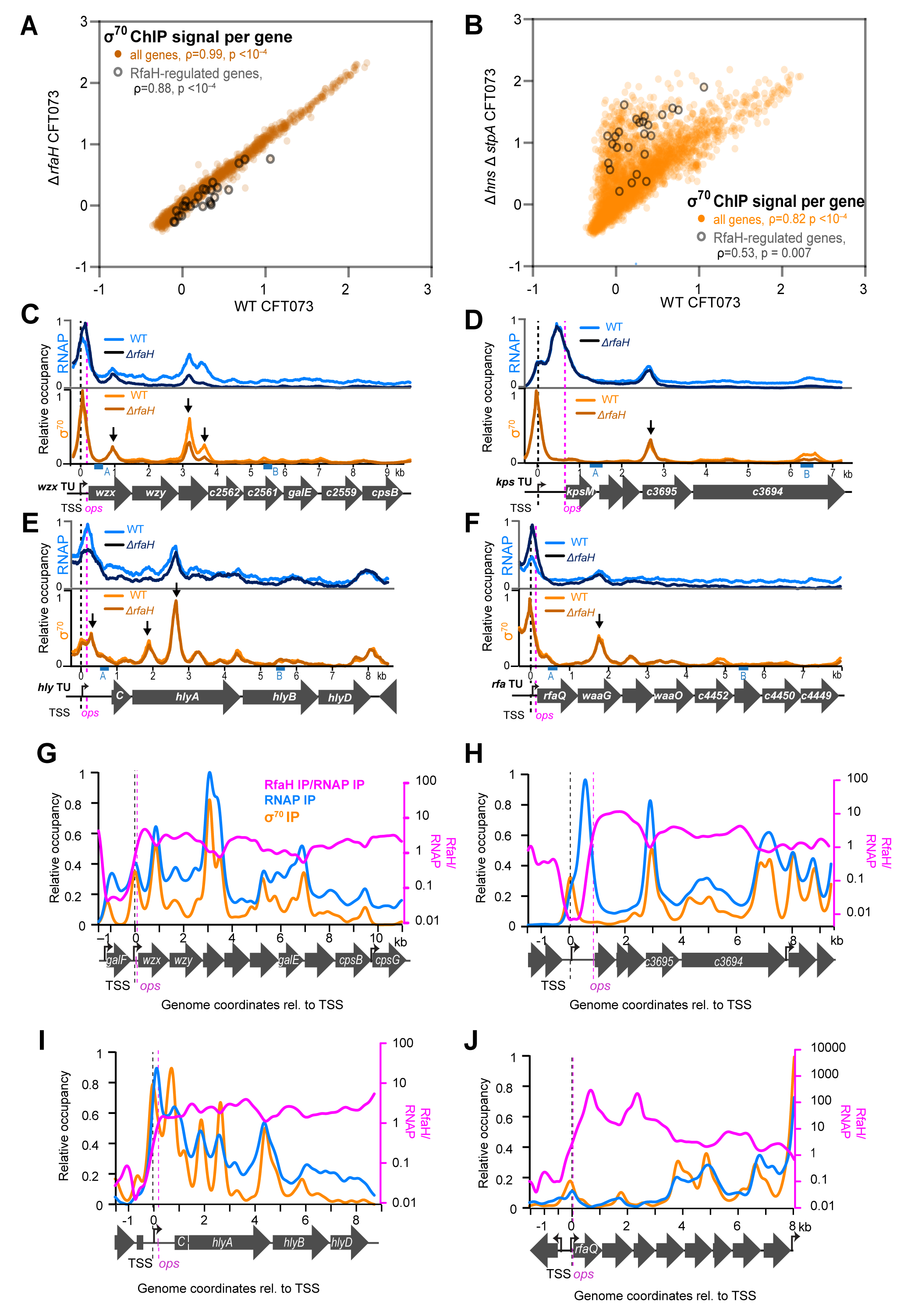

Supplement: FIG S5 [file mbio.02662-22-s0005.tif]

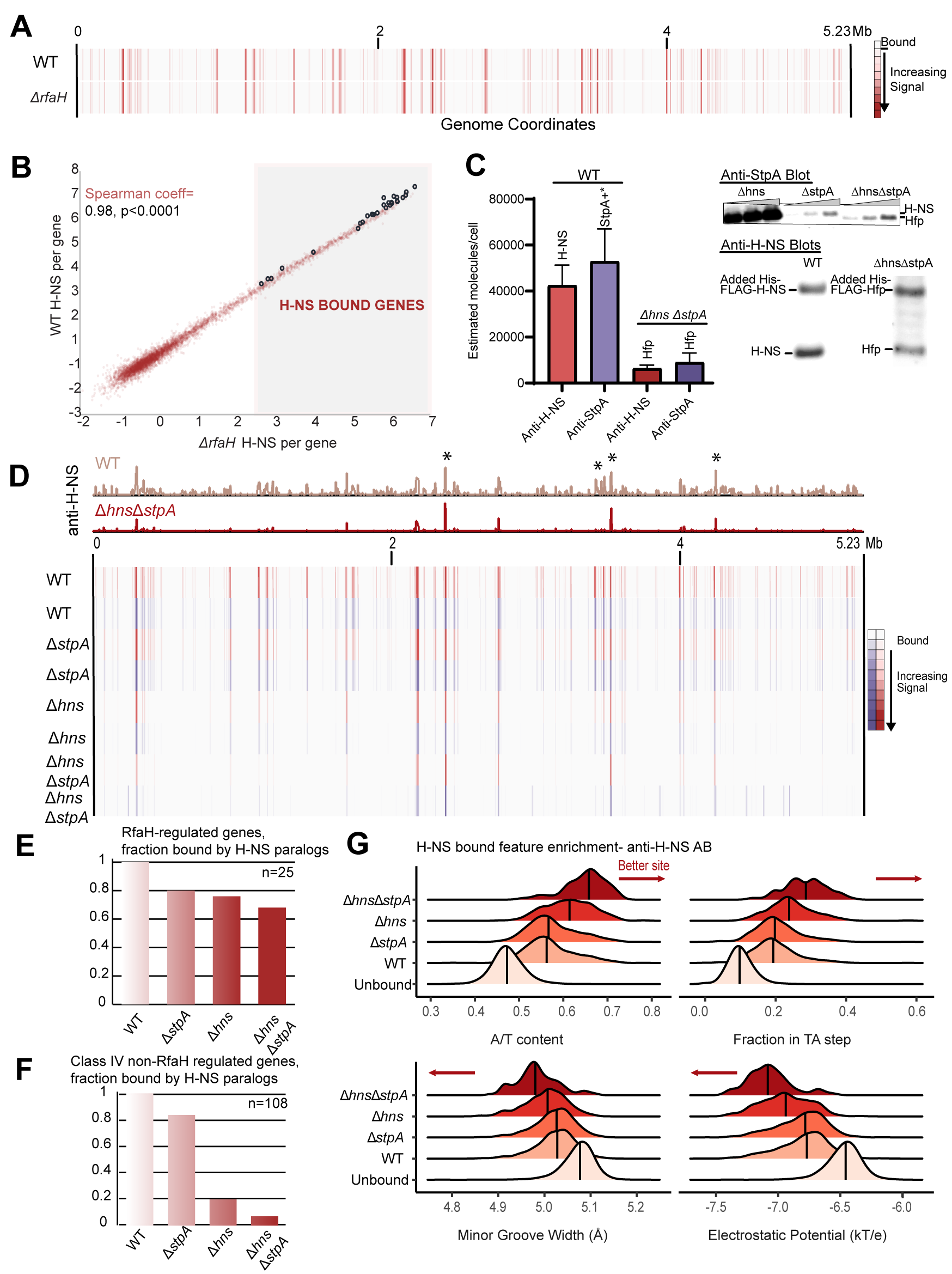

Supplement: FIG S6 [file mbio.02662-22-s0006.tif]
