# Supplementary material for: RfaH Counter-Silences Inhibition of Transcript Elongation by H-NS–StpA Nucleoprotein Filaments in Pathogenic Escherichia coli
Source: mBio. 2022 Oct 20;13(6):e02662-22. doi: 10.1128/mbio.02662-22 (PMC9765446; doi:10.1128/mbio.02662-22)
Supplement: Text S1 [file mbio.02662-22-s0007.pdf]

Extended methods for Hustmyer *et al.*

DNA methods and Reagents

PCR and restriction digests were performed using Q5 HF polymerase, OneTaq, and restriction enzymes from New England Biolabs. Oligonucleotides and gene blocks (Table S1) were obtained from IDT (Coralville, IA). Sequencing of PCR products and plasmids (Table S2) was obtained from QuantBio (Beverly, MA) or Functional BioSciences (Madison, WI). Chemicals and reagents were obtained from Sigma-Aldrich, Thermo Fisher, or Millipore Sigma unless specified otherwise.

CFT073 Strain Construction

A modified recombination protocol using *E. coli* CFT073 (WAM4505) harboring λ*red* recombination machinery (WAM4507) was used to make gene deletions, similarly to previous descriptions (1, 2). Briefly, genes were deleted on the chromosome in-frame and replaced with a kanamycin-resistance cassette that was amplified from pKD4 with 5′ and 3′ segments that could base-pair upstream or downstream, respectively, of the gene to be deleted. PCR products to delete H-NS, StpA, and RfaH were amplified using oligos #2945 and #2946, #2947 and #2948, and #14392 and #14393, respectively. After construction, the deletions were transduced into a clean WT WAM4505 background with CFT073-specific phage ΦEB49 (3) and these transduced strains were used in ChIP-seq experiments. To avoid suppressor mutations, *Δhns*::*kan* derivatives were freshly transduced using ΦEB49 prior to each cell collection. Other antibiotic-resistance cassettes in strains WAM5678 and WAM5701 were removed using pCP20 prior to *Δhns*::*kan* transduction to generate double and triple deletion strains. Deletions were confirmed by colony PCR using primers flanking the deletion and internal to the deleted gene, Sanger sequencing of the flanking amplicon, and by verifying lack of read coverage for the gene in the ChIP input sample data (see Bioinformatics Analysis). Cultures grown for cell collection for ChIP-seq were not supplemented with antibiotics. Strains used for intermediate construction and well as final chromosomal deletions used for ChIP-seq are listed in Table S3.

Plasmid preparation

An expression plasmid for StpA lacking linkers and epitope tags (pWTStpANat) was constructed by Gibson assembly using NEB HiFi reagents. The *stpA* coding sequence was amplified from *E. coli* K-12 strain MG1655 genomic DNA using primers #11783 and #11784 (Table S1) and the

pET21d backbone was amplified from pWTHNS using primers #11781 and #11782 before assembly.

StpA purification for antibody generation

StpA lacking tags was purified as previously described (4, 5) with several major modifications. Briefly, *hns::kan* (6) was transduced using P1 into BL21 λDE3 co-transformed with pWTStpANative and pHiC. Cells were grown in 2 L LB supplemented with ampicillin (100 µg/mL) and gentamycin (10 µg/mL) at 37 °C to an apparent OD_{600} of ~0.2, at which point the incubator temperature was decreased to 25 °C. At an apparent OD_{600} of ~0.4, the cells were supplemented with carbenicillin (250 µg/mL). StpA expression was then induced by addition of isopropyl B-D-1-thiogalactopyranoside (IPTG) to 0.5 mM and incubation for 18 h at 25 °C. Cells were split into 1 L volumes and centrifuged at 3000 × *g*, 30 minutes, 4° C. Each 1 L cell pellet was resuspended in 15 mL lysis buffer (0.2 M NaCl, 50 mM Tris-HCl pH 7.5, 5% glycerol, 2 mM ethylenediaminetetraacetic acid (EDTA), 1 mM β-mercaptoethanol (β-ME), 1 mM dithiothreitol (DTT)) supplemented with 0.1 mg PMSF/mL phenylmethylsulfonyl fluoride (PMSF) and protease inhibitor cocktail (PIC; 31.2 mg benzamide/mL, 0.5 mg chymostatin/mL, 0.5mg leupeptin/mL, 0.1mg pepstatin/mL, 1 mg aprotonin/mL, 1 mg antipain/mL). Cells were lysed with 1.5 ng/mL lysozyme for 30 minutes followed by sonication for ~40 minutes at 25% duty cycle and power level 6. The lysate was cleared by centrifugation at 27,000 × *g*, 20 min, 4 °C. StpA was first enriched by precipitation of DNA-associated proteins with polyethyleneimine (PEI; 50% from Acros Organics–Thermo-Fisher). PEI (0.03 vol of a 10% w/v PEI solution) was added to the lysate with gentle stirring for 5 min at 4 °C (final 0.3% PEI). PEI 10% solution was prepared by dialysis overnight at 4 °C against 10 mM Tris-HCl pH 7.5, 100 mM NaCl, 5% glycerol, 0.1 mM EDTA. The precipitate was collected by centrifugation at 11,000 × *g*, 15 min, 4 °C. The pellet from 1 L cell culture was resuspended in 30 mL 50 mM Tris-HCl pH 7.5, 0.1 mM EDTA, 5% glycerol, 1 mM DTT, 0.75 M NaCl and collected by centrifugation at 11,000 × *g*, 15 min, 4 °C. StpA was precipitated with gentle stirring overnight with 0.4 g $AmSO_4$ powder/1 mL starting solution and collected by centrifugation at 27,000 × *g*, 15 min, 4 °C. The StpA pellet was resuspended in 20 mL 50 mM Tris-HCl, pH 7.5 and dialyzed in 3,500 MWCO tubing (Spectra/Por) against 1 L of no-salt precipitation buffer (50 mM Tris-HCl pH 7.5, 1 mM EDTA, 10% glycerol, 0.5 mM DTT) for 2 h and then overnight at 4 °C after the dialysis solution was replaced. StpA precipitated during the dialysis. The StpA precipitate

was collected by centrifugation at 8,000 × *g*, 15 min, 4 °C. The pellet was resuspended in 35 mL of Heparin binding buffer A (50 mM Tris-HCl pH 7.5, 1 mM EDTA, 10% glycerol, 0.3 M NaCl, 0.5 mM DTT) and was loaded onto a HiTrap Heparin HP column attached to an AktaPure (GE Healthcare) at a 0.5 mL/min flow rate as described previously (7). The column was washed with 30 mL of Heparin binding buffer A and eluted with a 0-100% gradient of Heparin binding buffer B (50 mM Tris-HCl pH 7.5, 1 mM EDTA, 10% glycerol, 1 M NaCl, 0.5 mM DTT) over 40 minutes at 1 mL/minute. Fractions containing StpA were pooled and dialyzed overnight with 3,500 MWCO tubing in no-salt precipitation buffer. Precipitated StpA was collected by centrifugation at 8,000 x *g*, 10 min, 4 °C, and resuspended in StpA storage buffer (50 mM Tris-HCl pH 7.5, 1 mM EDTA, 5% glycerol, 0.3 M NaCl). StpA purity was confirmed by sodium dodecyl sulphate-polyacrylamide gel electrophoresis (SDS-PAGE) and stored at –80 °C. StpA preps from multiple rounds of purification were pooled from overexpression in 12 L LB to generate 1.5 mg total of StpA for antiserum generation in rabbits by Covance (Denver, PA).

Antibodies and antibody purification

Monoclonals antibodies against σ^{70} (Biolegend, #663207) and RNAP (Biolegend, anti-β subunit, #663905) were used for σ^{70} and RNAP ChIP-seq. Polyclonal antibodies raised in rabbits against RfaH, H-NS, and StpA were used for RfaH, H-NS, and StpA ChIP-seq. RfaH antiserum was a gift from Irina Artsimovitch, Ohio State University, as described previously for RfaH ChIP-chip in MG1655 (8). H-NS antiserum was as described previously (9, 10). StpA antiserum was raised from purified StpA by Covance (Denver, PA).

H-NS (Harlan, Final bleed, ID R10041, Cat POL001) and StpA (Covance, Final bleed, WI631) antisera were pre-cleared by adsorption to cell powders produced from appropriate CFT073 deletion strains to reduce cross-reactivity. Briefly, 1 L cultures of WAM5676 and WAM5677 were grown to an apparent OD_{600} of ~0.4 in MOPS rich-defined medium (RDM) + 0.2% glucose (11) (Teknova) and collected by centrifugation at 4,000 × *g*, 15 min, 4 °C. The pellets were resuspended in 1 mL of cold 0.9% NaCl/1 g of pellet to induce gentle lysis for 5 min on ice. The solution was then mixed with a 4:1 ratio of acetone (chilled at –20 °C) to resuspension and then incubated on ice for 30 min with occasional vortexing. The acetone precipitate was collected by centrifugation at 10,000 × *g*, 10 min, 4 °C. The pellet was resuspended with half the original volume of acetone, incubated for 10 min at 0 °C, and collected

by centrifugation at 10,000 × *g*, 10 min, 4 °C. The pellet was dried overnight at 0 °C into a powder. The powder was resuspended in 10 mL of 1X IP Buffer (100 mM Tris, pH 8.0, 300 mM NaCl, 1% TritonX-100, 1 mM PMSF). Antiserum was immunodepleted upon addition of 2 mL of H-NS antiserum, as used in (9, 10) in WAM5676 and 2 mL of StpA antiserum in WAM5677. The resuspension was rocked gently for 30 minutes at 4° C to allow collection of antiserum to non-target epitopes. The remaining non-bound antibodies were collected from the supernatant after centrifugation at 10,000 × *g*, 10 min, 4 °C and stored at –80 °C. Specificity and success of purification of H-NS and StpA antisera was assessed by western blot on nitrocellulose membrane comparing antisera specificity before and after immunodepletion using whole-cell lysates of WAM4505 (WT), WAM5676 (Δ*hns*), WAM5677 (Δ*stpA*), and Δ*hns*Δ*stpA* grown in MOPS RDM + 0.2% glucose and harvested at an apparent OD_{600} of ~0.4 (Fig. S1F, G).

Chromatin-Immunoprecipitation sequencing

Cell collection and crosslinking

Cells were harvested and processed for ChIP-seq essentially as previously described (12), with minor modifications. CFT073 strains were grown in MOPS RDM + 0.2% glucose. We used RDM because CFT073 Δ*hns*Δ*stpA* exhibited a prohibitively slow growth rate in MOPS minimal media. Strains were grown in 400 mL of MOPS RDM in 2 L Erlenmeyer flasks aerobically at 37 °C with shaking at 140 rpm until mid-exponential phase (apparent OD_{600} ~0.4). Crosslinking was then initiated by addition of 10.4 mL formaldehyde (37% in H_2O) and 4 mL 1 M sodium phosphate, pH 7.6 without removing the culture from the shaker. Crosslinking was continued for 5 min at 37 °C with continuous shaking at 140 rpm. Crosslinking was then quenched by addition of glycine to 500 mM and placement of flasks in an ice-water slurry for 30 min with occasional rotation by hand. Cells were recovered by centrifugation at 3500 × *g*, 10 min, 4 °C and washed 3 times with 800 mL of phosphate-buffered saline (PBS; 137 mM NaCl, 2.7 KCl, 10 mM Na_2HPO_4, 1.8 mM KH_2PO_4). The resulting pellet was resuspended in 8 mL PBS and transferred in eight 1 mL aliquots to 1.5 mL microfuge tubes. Cells were collected by centrifugation at 3500 × *g*, 10 min, 4 °C, washed once with 1 mL PBS, and then centrifuged once more at 3500 × *g*, 10 min, 4 °C. The resulting cell pellets were frozen and stored at –80° C. One cell pellet corresponding to 50 mL of original culture volume was used for each IP. Crosslinked cells from

the same cultures were used for parallel IPs for each replicate (e.g., for H-NS, StpA, RNAP, RfaH, and σ^{70}).

Immunoprecipitation

Crosslinked cell pellets from 50 mL of culture were thawed and resuspended in 500 µL IP buffer (100 mM Tris, pH 8.0, 300 mM NaCl, 1% TritonX-100, 1 mM Pefabloc SC [Millipore Sigma]) and sonicated using a Misonix Ultrasonic Liquid Processor (Model No. S-4000) for 16–60 minutes at 60% power with 10-sec on–10-sec off cycles, to generate DNA fragments of ~300–500 bp. RNase A was then added to 2 µg/mL and the sonicates were incubated for 1 h at 4° C with rotation on an microfuge tube rotator at ~8 rotations per min. To generate IP input controls, 1/10th (~50 µL) of the resulting lysate was removed to a separate tube. The resulting input control sample was incubated overnight at 4 °C with rotation on a microfuge tube rotator at ~8 rotations per min with 30 µL of magnetic beads (NEB) coated with Protein A or Protein G (for comparison to monoclonal antibody or polyclonal antibody IPs, respectively) and 700 µL of IP buffer. The remaining lysate was pre-cleared for 3 h at 4 °C by incubation with 30 µL of the appropriate Protein A or G magnetic beads to remove protein–DNA complexes that bound beads non-specifically and an addition of 250 µL IP buffer to retain beads in solution (750 µL total volume). The beads were removed using a magnetic microfuge-tube stand (NEB) and the pre-cleared lysate supernatant was then incubated after addition of antibody (2 µL monoclonal antibodies for σ^{70} and RNAP, 10 µL polyclonal antisera for RfaH, or 10 µL pre-adsorbed polyclonal antisera for H-NS and StpA) for 17–19 hours at 4 °C at ~8 rotations per min on a microfuge tube rotator. Antibody–protein–DNA complexes were then recovered using the magnetic stand and washed once with 1 mL LiCl solution (250 mM LiCl, 100 mM Tris-HCl pH 8, 2% TritonX-100), twice with 1 mL 600 mM NaCl solution (100 mM Tris-HCl pH 8, 600 mM NaCl, 2% TritonX-100), twice with 1 mL 300 mM NaCl (100 mM Tris-HCl pH 8, 300 mM NaCl, 2% TritonX-100), and twice with 1 mL TE (10 mM Tris-HCl pH 8, 1 mM EDTA). Protein–DNA complexes were then eluted from beads by addition of 100 µL ChIP elution buffer (50 mM Tris-HCl pH 8.0, 10 mM EDTA, 1% SDS) and incubation for 1 h at 65° C. The beads were then removed using the magnetic stand and crosslink reversal was completed by incubation for 18 h at 65° C. DNA was then recovered using Qiagen QIAquick PCR purification reagents and eluted in 58 µL of the Qiagen-supplied elution buffer. The DNA concentration was assessed

using a QuBit dsDNA HS assay (ThermoFisher) on a QuBit 3.0 fluorimeter and then stored at – 20 °C.

Input and no-antibody controls were generated similarly. Briefly, following overnight incubation with beads, ~750 µL of input supernatant was removed from no-antibody beads using a magnetic stand. Input supernatant was incubated at 65° C overnight after addition of 100 µL ChIP elution buffer to reverse protein–DNA crosslinks. Input DNA was checked on a 1.5% agarose DNA gel to confirm proper sonication and shearing (typically 200–1500 bp with median 200–600). The remaining beads ("no antibody") were processed and eluted as described for ChIP samples. No DNA was detected in the "no antibody" controls using the QuBit assay so only the ChIP and input samples were used to prepare libraries for DNA sequencing.

IPs, inputs, and no-antibody controls were assessed for enrichment using ChIP-qPCR on a QuantStudio3 using PowerUp SYBR master mix (Thermo Fisher) before library preparation.

Paired-end libraries for DNA sequencing were prepared using the NEBNext Ultra II DNA library reagents with NEBNext sample purification beads according to the manufacturer's protocols without size selection. Quality and size ranges of libraries was determined using HS DNA Screentape (Agilent) on a TapeStation 4200 system (Agilent). Libraries were sequenced on Illumina MiSeq or NovaSeq 6000 sequencers.

Bioinformatic analysis

ChIP-seq analysis

All ChIP-seq analysis were performed using an open-source-software-based ChIP-seq pipeline (https://github.com/cmhustmyer/2022_hustmyer). The pipeline manages computational jobs using Snakemake (5.24.2) (13) and maintains the relationship between samples and their metadata using peppy (14). Paired- and single-end reads were trimmed of adapter sequences using CutAdapt version 2.10 (15) with parameters '-a AGATCGGAAGAGCACACGTCTGAACTCCAGTCA -A AGATCGGAAGAGCGTCGTGTAGGGAAAGAGTGT' for paired-end samples and '-a AGATCGGAAGAGCACACGTCTGAACTCCAGTCA' for single-end samples. Quality trimming was performed using trimmomatic (version 0.39) (16) with parameters "LEADING:3 TRAILING:3 SLIDINGWINDOW:4:15" with the respective PE or SE mode for paired-end and single-end samples. The reads were aligned to the respective genome (Refseq NC_004431.1 for

CFT073 or NC_000913.3 for RL3000) using bowtie2 (version 2.4.2) (16) with parameters "--end-to-end --very-sensitive --phred33". For deletion strains, reads were first aligned to the WT genome to confirm lack of read coverage over the deleted gene, and then aligned with each deleted gene masked. Read coverage in five bp bins was scaled to the median coverage over all bins for each sample (17). Read coverage was calculated using deepTools (version 3.5.0) (18) with the parameters "--binSize 5 --samFlagInclude 66 --extendReads" for paired end samples and " --binSize 5" for single end samples. Normalized coverage based on the input (IP/Input) or $\log_2$(IP/input) was calculated with custom python scripts. Read quality control was performed using fastqc version 0.11.8 (https://www.bioinformatics.babraham.ac.uk/projects/fastqc/) and multiqc version 1.11.0 (18).

RfaH-bound regions (Dataset 2) were identified using the following steps. First, enriched RfaH regions were called in WT, *ΔrfaH*, and *ΔhnsΔstpA* using macs2 (version 2.2.7.1) (19) with the parameter "--broad" using the corresponding input as a control sample. Each region was then verified in WT or *ΔhnsΔstpA,* using two criteria: an *ops* site was present within the region and the region was less than 1.5-fold enriched over background in *ΔrfaH* (i.e., the signal significantly decreased or was not present in the RfaH *ΔrfaH* control ChIP).

ChIP signal averages per gene, window, and TU were calculated using custom python scripts. Biological replicates with a Pearson correlation coefficient of least ~0.94 at IP/input signal per gene were used to calculate average ChIP signals after normalization. Gene annotations were parsed from GenBank NC_004431.1 and NC_000913.3 using custom python scripts. Genes for which reads were absent in input or IP for more than 20% of its length were excluded from analyses.

To check for any undesired genetic changes that might have arisen during cell growth, input sample sequences were compared to the NCBI reference NC_004431.1 sequence file for CFT073 (20) using breseq (21). We discovered many (>350) sequence differences that were common to all our CFT073 strains relative to the NC_004431.1 sequence (Dataset S1G). We suspect most if not all represent errors in the NCBI record. We found a small number (12) of sequence variations that were present in only one or a few of our CFT073 lineages or datasets

(Dataset S1H). None of these 12 sequence differences affected genes encoding RNAP subunits, H-NS, StpA, RfaH, NusG or σ^{70}.

Raw and processed reads from this work are deposited at GEO with accession number GSE212064. All input files, custom scripts, and pipeline definition code required to re-create the analysis described here is available at: https://github.com/cmhustmyer/2022_hustmyer. H-NS and StpA Myc-tagged ChIP-exo data (22) (GEO GSE181767) were also analyzed using this pipeline.

RNA-seq analysis to define transcription units

To define transcription units (TUs) in WT CFT073, we analyzed CFT073 RNA-seq data collected from cells grown in M9 minimal media supplemented with 0.2% glucose at mid-exponential phase (23) (GEO GSE122296, samples GSM3463591 and GSM3463592). TUs were inferred from RNA-seq data using Rockhopper (24). Transcript isoforms that were within a Hausdorff distance of 100 bp were merged into a single transcript with boundaries set to the maximum 5′ and 3′ ends of the set of similar transcripts. Because only WT RNA-seq data was available, TUs that we hypothesized are not transcribed in WT (e.g. *wza*) could not be defined using Rockhopper. Relevant TU boundaries and approximate TSSs from apparent TU boundaries and σ^{70} peaks are noted in figure legends and in Dataset S2.

RNAP occupancy normalization

To compare RNAP occupancies across strains for which major genome-scale differences in transcription patterns are not expected (e.g,. to compare WT and *ΔrfaH*, where RfaH targets only 4 sites in the entire WT CFT073 genome), we scaled RNAP ChIP signals linearly between the maximal signal for bound RNAP (assigned as 1) and the background signal (assigned as 0) for genes at which RNAP was bound only non-specifically, as described previously for RNAP ChIP-chip data (25) with minor modification. RNAP binds non-specifically to non-transcribed DNA (26), resulting in background ChIP signal (Fig. 1E). To define the background RNAP ChIP signal, we averaged ChIP signals for the 20 genes with lowest average RNAP ChIP signal in each replicate and assigned as 0 RNAP occupancy for that replicate. In our analyses of CFT073 RNAP ChIP-seq (Dataset S1), ~9% of coding genes were indistinguishable from this background signal. This untranscribed percentage of the CFT073 genome is similar to the 7% of genes found as RNAP background for MG1655 (25). Maximal RNAP occupancy occurs on rRNA and tRNA

genes (27). Therefore, to assign the RNAP occupancy value of 1, we averaged the RNAP ChIP signals for 20 pre-determined tRNA genes, with had amongst the highest (top 50) average RNAP ChIP signal in each replicate and each strain (see https://github.com/cmhustmyer/2022_hustmyer). We excluded rRNA genes from this analysis due to complications mapping reads to the 7 homologous rRNA operons. This method of RNAP occupancy scaling allows us to compare RNAP occupancy across strains (e.g., WT *vs. ΔrfaH, ΔstpA vs. ΔstpAΔrfaH,* and *ΔhnsΔstpA vs. ΔhnsΔstpAΔrfaH*).

Global occupancy scaling was performed using custom python scripts in 5-bp windows available at https://github.com/cmhustmyer/2022_hustmyer.

Traveling ratios

Traveling ratio (TR) calculations were performed as described previously (25, 28), with minor modifications (Fig. 3F and Dataset S3). The TR for RfaH-regulated genes was defined as the ratio of average RNAP occupancy in a 300 bp window 5.4-5.8 kb downstream from the *ops* to the average RNAP occupancy 400-700 bp downstream from the *ops*. These windows were chosen to capture the RfaH-based effects on elongation. A window too near the TSS included signal from promoters that was convoluted with signal from elongating RNAP; 0.4 kb downstream of *ops* avoided signals from promoters so a meaningful elongation traveling ratio could be calculated. Four non-RfaH or H-NS regulated TUs were also analyzed as controls (Dataset S3, Fig. S4A-B). For the controls, the upstream 300-bp window was centered 0.3 kb from the TSS for each TU and the downstream window was either centered 5 kb from the 5′ 0.3 kb window or, if the TU was not long enough, the downstream-most 0.3 kb of the TU. RfaH-dependent TRs (RTRs) were calculated by dividing the TR for a Δ*rfaH* strain by the TR for the corresponding *rfaH*$^+$ strain. All TR data are included in extended dataset S3.

H-NS and StpA occupancy normalization

To compare H-NS and StpA distributions in various strains, we used an occupancy normalization similar to the RNAP occupancy normalization. Each ChIP dataset was normalized to an appropriate input dataset (e.g., WT H-NS IP was normalized to WT input, H-NS IP in *ΔstpA* was normalized to the *ΔstpA* input). Average signals per gene or window were calculated using two or three biological replicates. These average signals were then linearly scaled between 0 and 1, where 0 was the average of signals for the 20 genes with lowest signal in the H-NS IP and 1 was

the average of the 20 genes with highest signal for each IP. The average occupancy-normalized H-NS and StpA signals were then calculated per gene (Fig. 6) or per 1.4 kb window (Fig. S6). The cut-off for H-NS bound genes in WT CFT073 was determined by an apparent inflection point in a cumulative plot of average H-NS ChIP-signal per gene (Fig. S1A). This yielded a cut-off of 0.07 times the maximal average H-NS ChIP signal per gene and the set of genes shown in Fig. 6C. To determine the subset of these genes scored as still bound in *ΔrfaH*, *ΔstpA, Δhns, ΔhnsΔstpA* strains (Dataset 1, Fig. 6C), we used a cut-off that was 0.07 times the maximal average ChIP signal per gene for that strain. For both WT and deletion strain determinations, the maximal average ChIP signal per gene was set as the average for the top 20 genes.

Relative ChIP occupancy for local transcription units

To compare ChIP signals for different targets within the same strain (*e.g.* Fig. 2, Fig. S3, Fig. S5G-J), ChIP data were normalized to local maximal and minimal signals. For Fig. 2, raw ChIP signals (median-read normalized) from 1800 nt US and DS of 5′ and 3′ ends of predicted TSS were averaged across replicates. Each IP was then individually normalized within the TU window, using the average of the five lowest 5-bp window signals as background (set to 0) and the average of the five 5-bp window highest signals set to 1. Sequential rolling averages over 25 bp windows (at least 5 times) were then used to smooth signals, which also had the consequence of causing the highest peaks to be somewhat below 1. For RfaH/RNAP ratios in Figs. S5C-E and S5G-J, signal was log-scaled.

Ortholog analysis

Orthologous gene pairs between CFT073 (NC_004431.1) and MG1655 (NC_000913.3) were identified using https://github.com/ridgelab/JustOrthologs (29) with default parameters. Orthologous pairs and lineage-specific genes were cross-referenced against OrthologeDB 2.1 (https://orthologedb.ca/?page=index) (30) for MG1655 and CFT073. Genes pairs for which results conflicted were excluded from analysis (extended dataset 1).

ops *motif alignment and genome-wide calling*

ops motifs from the eight bona-fide RfaH-regulated TUs (extended dataset S2) were aligned using SnapGene (Dotmatics, Boston, MA; MUSCLE vsn 3.8.1151 with default settings) (31). The *ops* sequence logo was generated using weblogo (https://weblogo.berkeley.edu). A search for *ops* motifs genome-wide in CFT073 was performed using FIMO motif scanning

(https://meme-suite.org/meme/tools/fimo) using the *ops* sequence logo and a fasta sequence filed generated from GenBank NC_004431.1. Both strands were scanned using a match *p*-value of < 1E-6. All *ops* sites called are listed in Dataset 2 and cataloged by their strand orientation, genomic location, and whether RfaH signal was enriched over input using macs2.

H-NS sequence, structure, and electrostatic parameter predictions

H-NS sequence, structure, and electrostatic features (Fig. S6G) were predicted using custom scripts as described (32). Analysis code is available at https://zenodo.org/record/6498897.

Data visualization

ChIP traces were generated using the Integrative Genomic Viewer (IGV, Version 2.12.3) (33). Data plots were prepared using GraphPad Prism for Windows 9, Excel, or R (version 4.0.5) (34). Heat maps were generated using the Interactive CHM builder (MD Anderson Cancer Center, University of Texas) (35).

Quantitative western blots

Quantitative western blots were performed as previously described (26) with minor modifications using *in vitro* synthesized H-NS, StpA, and Hfp as standards.

Plasmid preparation for* in vitro *synthesis of H-NS, StpA, and Hfp

Expression plasmids for *in vitro* protein synthesis were constructed from synthetic DNA fragments (IDT) encoding a T7 RNAP promoter, CFT073 *hns*, *stpA*, or *hfp*, a C-terminal 3X FLAG epitope tag, and a 3′ stem-loop RNA structure (Tables S1, S2). DNAs were 5′-phosphorylated using T4 polynucleotide kinase (NEB) and then blunt-end ligated into pSMART HCKan (Lucigen) to yield pCMH01-03 (Table S2). Several sequence modifications were made to aid protein expression. A 6X-His tag was added after the 3X FLAG tag using primers #14968 and #14969 by Q5 QuickChange (NEB) to generate pCMH04-06 (Table S2). Each coding sequence flanked by C-terminal 3X-FLAG and 6X His tags was PCR amplified using primers #15085 (H-NS), #15087 (StpA), and #15089 (Hfp) with #15086 to amplify the C-terminal His region (Table S1). A backbone fragment that contained a full T7 terminator was amplified from pT7_terminator (NEB, Table S2) using primers #15083 and #15084. The purified PCR products were Gibson assembled to generate pCMH008-010 (Table S2) that were used for *in vitro* protein synthesis.

In vitro *protein synthesis for western blot analysis*

C-terminal 3XFLAG and 6XHis-tagged H-NS, StpA, and Hfp were synthesized for western blot analysis using the PUREfrex 2.0 reconstituted cell-free protein synthesis reagents (Cosmo Bio USA) (36). pCMH08-10 (~30 ng) were used as templates. Proteins were synthesized for 4 hours at 37 °C following the manufacturer's protocol. Protein synthesis was confirmed using SDS-PAGE. Protein concentrations of *in vitro* synthesized proteins were estimated by Coomassie staining after SDS-PAGE compared to a purified H-NS standard of known concentration quantified using the QuBit protein assay (ThermoFisher) on a QuBit 3.0 fluorimeter (5).

Measurement of number of cells and total cellular protein

CFT073 or RL3000 strains were grown in MOPS rich–defined media. Cell pellets from one mL of culture were isolated by centrifugation at apparent OD_{600} ~0.4 and stored at –80 °C. Cell numbers were estimated from colony-forming units on LB plates incubated at 37 °C. To generate cell lysates, pellets were resuspended in 1 mL of 1X PBS, combined with 0.1 mL of 0.15% deoxycholic acid, and incubated at room temperature for 10 min. Trichloroacetic acid (TCA) (111 µL 50% w/v) was then added and the mixture was incubated on ice for 30 min. The precipitate was collected by centrifugation at 22,000 × *g*, 30 min, 4 °C. The precipitate was resuspended in 60 µL a mixture of 0.1M Tris HCl (pH 6.8) and 4% SDS and held for 10 min at 100 °C. The Pierce BCA protein assay (Thermo Scientific) was used to generate a standard curve to estimate total cellular protein concentration according to the manufacturer's protocol. After combining with assay reagents, standards and unknown proteins were measured at OD_{562} on a Tecan M1000 Infinite plate reader.

Preparation of whole-cell extracts and western blots

Pellets were lysed and TCA precipitated as described above, centrifuged, then resuspended in 60 µL 2X SDS sample buffer (0.125 Tris-HCl, pH 6.8, 4% SDS, 20% glycerol, 0.02% bromophenol blue, 1.43 M ß-mercaptoethanol). Excess TCA was neutralized using puffs of NH_3 vapor from a Pasteur pipette after which proteins were denatured by incubation for 10 min at 100 °C. To measure cellular proteins levels, *in vitro* synthesized H-NS-Flag-His, StpA-Flag-His, or Hfp-Flag-His were mixed at increasing concentrations with constant amounts of cellular lysates and subjected to SDS-PAGE and western blotting to generate standard curves. Samples were loaded

onto a Bolt 12% Bis-Tris Plus polyacrylamide gel (Invitrogen) and transferred to a Nitrocellulose blotting membrane (0.2 µm, Amersham) using a PowerBlotter Station (Invitrogen) under low molecular weight settings for 7 min. For WT CFT073, *in vitro* synthesized H-NS and StpA were titrated in each lane to measure H-NS and StpA levels, respectively, whereas *in vitro* synthesized Hfp was used to measure cellular Hfp levels in *ΔhnsΔstpA.* Blots were probed with 1:5,000 dilutions of purified H-NS or StpA antisera and then incubated with 1:10,000 dilution of IRDye 800CW goat anti-rabbit secondary (Li-Cor). Blots were visualized on a ChemiDoc MP (Bio-Rad) using IRDye 800 CW settings. Signals of estimated amounts of *in vitro* synthesized proteins were fit to a linear line using ImageLab software (Bio-Rad) and used to estimated cellular concentrations of H-NS, StpA, and Hfp. All experiments were repeated at least three times with three biological replicates for CFT073 strains.

Table S1. Oligonucleotides used in this study.

Primer Number	Description	Sequence
CH14392	Lambda-red recombineering, 5′ CFT073 RfaH to amplify pKD4	5′-gccaccacggatgccaatgtcaaaacactgtttgggattgcgttttagaggtgtaggctggagctgcttc
CH14393	Lambda-red recombineering, 3′ CFT073 RfaH to amplify pKD4	5′-aaagcttttgctatccttgcgccccgattaaacggataagagtcattatgatgggaattagccatggtcc
CH11783	Amplify *stpA* MG1655 Fwd	5′-ttgttagcagccggatctcagatcaggaaatcgtcgag
CH11784	Amplify *stpA* MG1655 Rev	5′-ctttaagaaggagatataccatgtccgtaatgttacaaag
CH11781	Amplify pET21d_rev	5′-tgagatccggctgctaac
CH11782	Amplify pET21d_rev	5′-ggtatatctccttcttaaagttaaacaaaattatttctagaggggaattgttatccgc
WAM2945	Lambda-red recombineering, 5′ CFT073 H-NS to amplify pKD4	5′-caacaaaccaccccaatataagtttgagattactacaatggtgtaggctggagctgcttc
WAM2946	Lambda-red recombineering, 3′ CFT073 H-NS to amplify pKD4	5′-gatggcgggatttttagcatgtgcaatctacaaaagattacatatgaatatcctccttag
WAM2947	Lambda-red recombineering, 5′ CFT073 StpA to amplify pKD4	5′-atactttttgttttgggttaaaaggttttctttattatggtgtaggctggagctgcttc
WAM2948	Lambda-red recombineering, 3′ CFT073 StpA to amplify pKD4	5′-ccggacgcgccatagcagcggcatccggcctcagtaattacatatgaatatcctccttag
CH14045	Verify *stpA* deletion, anneals in-frame of *stpA* in CFT073, Fwd	5′-gcgaattctccattgacgtt
CH14046	Verify *stpA* deletion, anneals in-frame of *stpA* in CFT073, Rev	5′-ctgaccggtccaggttttag
CH14030	Verify *stpA* deletion, flanking primer, anneals 250 nt upstream of *stpA* in CFT073, Fwd	5′-tcactttggttttgtcaggc
CH14031	Verify *stpA* deletion, flanking primer, anneals 250 nt downstream of *stpA* in CFT073, Rev	5′-tcccaacccttgccg
CH14020	Verify *hns* deletion, anneals in-frame of *hns* in CFT073, Fwd	5′-atgagcgaagcacttaaaattctg
CH14044	Verify *hns* deletion, anneals in-frame of *hns* in CFT073, Rev	5′-ttattgcttgatcaggaaatcgtc
CH14024	Verify *hns* deletion, flanking primer, anneals 250 nt upstream of *hns* in CFT073, Fwd	5′-agcctacgattatctcccc

CH14025	Verify *hns* deletion, flanking primer, anneals 250 nt downstream of *hns* in CFT073, Rev	5′-aataaattaggttacatgcaggcc
CH14016	Verify *rfaH* deletion, anneals in-frame of *rfaH* in CFT073, Fwd	5′-atgcaatcctggtatttactgtactg
CH14017	Verify *rfaH* deletion, anneals in-frame of *rfaH* in CFT073, Rev	5′-ttagagtttgcggaactcgg
CH14015	Verify *rfaH* deletion, flanking primer, anneals 250 nt upstream of *rfaH* in CFT073, Fwd	5′-ccaggtgcggatccac
CH14013	Verify *rfaH* deletion, flanking primer, anneals 250 nt downstream of *rfaH* in CFT073, Rev	5′-ttgcgatgaacgacg
CH14014	Verify *rfaH* deletion sanger sequencing primer, anneals 100 nt upstream of *rfaH* in CFT073, Rev	5′-agcgtgaactctgacgg
CH14012	Verify *rfaH* deletion sanger sequencing primer, anneals 100 nt downstream of *rfaH* in CFT073, Rev	5′-gccccatattttgcaacgtatt
CH14022	Verify *hns* deletion sanger sequencing primer, anneals 100 nt upstream of *hns* in CFT073, Fwd	5′-acaattttgaattccttacattcctg
CH14023	Verify *hns* deletion sanger sequencing primer, anneals 100 nt downstream of *hns* in CFT073, Rev	5′-gcaatcgacgccgtt
CH14028	Verify *stpA* deletion sanger sequencing primer, anneals 100 nt upstream of *stpA* in CFT073, Rev	5′-gctgaaataatctcgtgcagg
CH14029	Verify *stpA* deletion sanger sequencing primer, anneals 100 nt downstream of *stpA* in CFT073, Rev	5′-ttttgtggaatggcaagattgt
CH13659	Amplify *bglG* in CFT073, use in ChIP-qPCR as positive control for H-NS, StpA; negative control for Beta, Fwd	5′-tatctcgctaactgaccattgc
CH13660	Amplify *bglG* in CFT073, use in ChIP-qPCR as positive control for H-NS, StpA; negative control for Beta, Rev	5′-aagcccacttcatctttcgg
CH13642	Amplify *atpH* in CFT073, use in ChIP-qPCR as negative control for H-NS, StpA; positive control for Beta, Fwd	5′-tatcgcagtttgtggtgagcaac
CH13643	Amplify *atpH* in CFT073, use in ChIP-qPCR as negative control for H-NS, StpA; positive control for Beta, Rev	5′-cacggcacgcaggtgaataaa
CH14368	Amplify upstream promoter region of *atpI* in CFT073, use in ChIP-qPCR as positive control for σ^{70} enrichment, Fwd	5′-gcaccgtataatttgaccgctt

CH14369	Amplify upstream promoter region of *atpI* in CFT073, use in ChIP-qPCR as positive control for σ^{70} enrichment, Rev	5′-tttcactcctgctcccttcg
CH14684	Amplify *lacZ* in CFT073, use in ChIP-qPCR as negative control for σ^{70} enrichment, Fwd	5′-gcataaaccgaccacgcaaatc
CH14685	Amplify *lacZ* in CFT073, use in ChIP-qPCR as negative control for σ^{70} enrichment, Rev	5′-ttcaccctgccacaaagaaacc
CH14968	QuickChange to add 6X His tag to C-terminus F	5'-caccaccacTAACTAGCATAACCCCTC
CH14969	QuickChange to add 6X His tag to C-terminus R	5'-atgatgatgTTTGTCGTCATCGTCTTTG
CH15083	Amplify NEB PureExpress control vector F	5'-TGAGGATCCCGGGAATTC
CH15084	Amplify NEB PureExpress control vector R	5'-ATGTATATCTCCTTCTTAAAGTTAAACAAAATTATTTC
CH15085	Amplify H-NS-3XFLAG-6XHis F	5'-tttaagaaggagatatacatATGTCCGTAATGTTACAAAG
CH15086	Amplify 6XHis R	5'-gagaattcccgggatcctcaGTTAGTGGTGGTGATGATG
CH15087	Amplify H-NS-3XFLAG-6XHis F	5'-tttaagaaggagatatacatATGAGCGAAGCACTTAAAATTC
CH15089	Amplify HFP-3XFLAG-6XHis F	5'-tttaagaaggagatatacatATGAGTGAAGCTCTTAAGG
	CFT073 *hns CDS* gBlock with C-terminal 3X FLAG (dsDNA)	5'-GCGAATTAATACGACTCACTATAGGGCTTAAGTATAAGGAGGAAAAAATATGAGCGAAGCACTTAAAATTCTGAACAACATCCGTACTCTTCGTGCGCAGGCAAGAGAATGTACACTTGAAACGCTGGAAGAAATGCTGGAAAAATTAGAAGTTGTTGTTAACGAACGTCGCGAAGAAGAAAGCGCGGCTGCTGCTGAAGTTGAAGAGCGCACTCGTAAACTGCAGCAATATCGCGAAATGCTGATCGCTGACGGTATTGACCCGAACGAACTGCTGAATAGCCTTGCTGCCGTTAAATCTGGCACCAAAGCTAAGCGTGCTCAGCGTCCGGCAAAATATAGCTACGTTGACGAAAACGGCGAAACTAAAACCTGGACTGGCCAGGGCCGTACTCCAGCTGTAATCAAAAAAGCAATGGATGAGCAAGGTAAATCCCTCGACGATTTCCTGATCAAGCAA

		GGTAGCGGTgactacaaagacca<br>tgatggcgactacaaagaccatg<br>atatcgactacaaagacgatgac<br>gacaaacatcatcatcaccacca<br>cTAACTAGCATAACCCCTCTCTA<br>AACGGAGGGGTTT
	CFT073 *stpA CDS* gBlock with C-terminal 3X FLAG (dsDNA)	5'-<br>GCGAATTAATACGACTCACTATA<br>GGGCTTAAGTATAAGGAGGAAAA<br>AATATGTCCGTAATGTTACAAAG<br>TTTAAATAACATTCGCACCCTCC<br>GTGCGATGGCTCGCGAATTCTCC<br>ATTGACGTTCTTGAAGAAATGCT<br>CGAAAAATTCAGGGTTGTCACTA<br>AAGAAAGACGTGAAGAAGAAGAA<br>CAACAGCAGCGTGAACTGGCAGA<br>GCGCCAGGAAAAAATTAGCACCT<br>GGCTGGAGCTGATGAAAGCTGAC<br>GGAATTAACCCGGAAGAGTTATT<br>GGGTAATAGCTCTGCTGCGGCAC<br>CACGCGCTGGTAAAAAACGCCAG<br>CCGCGTCCGGCGAAATATAAATT<br>CATCGATGTTAACGGTGAAACTA<br>AAACCTGGACCGGTCAGGGCCGT<br>ACACCGAAGCCAATCGCTCAGGC<br>GCTGGCAGAAGGTAAATCTCTCG<br>ACGATTTCCTGATCGGTAGCGGT<br>gactacaaagaccatgatggcga<br>ctacaaagaccatgatatcgact<br>acaaagacgatgacgacaaaTAA<br>CTAGCATAACCCCTCTCTAAACG<br>GAGGGGTTT
	CFT073 *hfp* gBlock with C-terminal 3X FLAG (dsDNA)	5'-<br>GCGAATTAATACGACTCACTATA<br>GGGCTTAAGTATAAGGAGGAAAA<br>AATATGAGTGAAGCTCTTAAGGC<br>ACTGAACAATATTCGTACACTTC<br>GCGCACAGGCTCGTGAAACAGAT<br>CTGGCAACTCTGGAAGAGATGCT<br>GGAAAAACTCACCACAATCGTTG<br>AAGATCGCCGTGAGGAAGAAAAT<br>TCAGCCCGTAAAGAACAAGAAGA<br>ACGTCAGGCTAAACTGGAAGCCT<br>TCCGCCAGAAATTGTTAGAAGAC<br>GGTATCGATCCTACAGAACTACT<br>CGCTTCAGTTGGTTCATCCCAGC<br>CTAAAACCAAATCAACTCGTGCT<br>CCTCGTCCTGCTAAATACAAATA<br>TACAGATGAAAACGGTAATGAGC<br>AGACTTGGACGGGTCAGGGCCGT<br>ACTCCTAAAGCAATCGCCGCTGC<br>TATCGAAGCTGGTAAGACACTGG<br>AAGACTTTGCTATCGGTAGCGGT<br>gactacaaagaccatgatggcga

		ctacaaagaccatgatatcgactacaaagacgatgacgacaaaTAACTAGCATAACCCCTCTCTAAACGGAGGGGTTT

Table S2. Plasmids used in this study. Sequences are available upon request.

Plasmid Name	Stock Number	Description	Reference
pWTHNS	5052, pBB15	*E. coli* H-NS with N-terminus 6xHis tag and TEV cleavage site downstream of IPTG inducible promoter in pET21d (+) backbone	(32)
pWTStpANative	5054, pBB17	*E. coli MG1655 stpA* coding sequence cloned without the His-tag downstream of IPTG inducible promoter in pET21d (+) backbone	*This study*
pHiC		Single-copy BAC plasmid carrying an engineered lacI^{q1} repressor allele that expresses approximately 170-fold more Lac repressor than the wild-type *lacI* gene. Isolated from Lucigen strain HI-Control 10G.	Lucigen
pKD46		Plasmid for RED-recombineering	(1)
pKD4		Parent plasmid containing Kan casette and FRT sites for used to amplify PCR products for lambda red-recombineering Wanner method	(1)
pCP20		Plasmid expressing Flp used to excise Kan marker in chromosome at FRT sites	(1)
pSMART HCKan		High copy number vector with kanamycin resistance marker for efficient blunt cloning of unstable sequences	Lucigen
pHNS_FLAG	6162, pCMH01	*E. coli* CFT073 *hns* coding sequence cloned His-tag downstream of T7 promoter in and upstream of C-terminal 3X-FLAG	*This study*
pStpA_FLAG	6163, pCMH02	*E. coli* CFT073 *stpA* coding sequence cloned His-tag downstream of T7 promoter in and upstream of C-terminal 3X-FLAG	*This study*
pHFP_FLAG	6164, pCMH03	*E. coli* CFT073 *hfp* coding sequence cloned His-tag downstream of T7 promoter in and upstream of C-terminal 3X-FLAG	*This study*
pHNS_FLAG_His	6177, pCMH04	*E. coli* CFT073 *hns* coding sequence cloned His-tag downstream of T7 promoter in and upstream of C-terminal 3X-FLAG and quick-change addition of 6X-C terminus His tag	*This study*

pStpA_FLAG_His	6178, pCMH05	*E. coli* CFT073 *stpA* coding sequence cloned His-tag downstream of T7 promoter in and upstream of C-terminal 3X-FLAG quick-change addition of 6X-C terminus His tag	*This study*
pHFP_FLAG_His	6179, pCMH06	*E. coli* CFT073 *hfp* coding sequence cloned His-tag downstream of T7 promoter in and upstream of C-terminal 3X-FLAG quick-change addition of 6X-C terminus His tag	*This study*
pT7_term		Control expression vector encoding DHFR downstream of T7 promoter and RBS and followed by full T7 terminator/ Provided in NEB PureExpress in vitro synthesis kit	NEB
pHNS_FLAG_His_Term	6183, pCMH08	E. coli CFT073 *hns* coding sequence with C-terminal 3X FLAG and 6X *his* cloned into NEBExpress_control plasmid backbone with T7 promoter and full T7 terminator	*This study*
pStpA_FLAG_His_Term	6184, pCMH09	E. coli CFT073 *stpA* coding sequence with C-terminal 3X FLAG and 6X *his* cloned into NEBExpress_control plasmid backbone with T7 promoter and full T7 terminator	*This study*
pHFP_FLAG_His_Term	6185, pCMH10	E. coli CFT073 *hfp* coding sequence with C-terminal 3X FLAG and 6X *his* cloned into NEBExpress_control plasmid backbone with T7 promoter and full T7 terminator	*This study*

Table S3. Strains used in this study.

**Strain Number**	**Description**	**Reference**
WAM4505	CFT073 WT Strain, used for ChIP-seq as "WT"	American Type Culture Collection # BAA-2503 and (20, 37)
WAM4507	CFT073 WT strain harboring pKD46 (lambda red-recombineering plasmid)	(1)
ΦEB49	CFT073 generalized transducing phage	(3)
WAM5676	*Δhns::kan* in WAM4505 (always freshly transduced), used for ChIP-seq a*s Δhns*	*This study*
WAM5677	*ΔstpA::kan* in WAM4505*, used for ChIP-seq as ΔstpA*	*This study*
WAM5678	*ΔstpA* excised of kan marker by pCP20 in WAM4505	*This study*
WAM5691	*ΔrfaH::kan* in WAM4505, used in ChIP-seq as *ΔrfaH*	*This study*
WAM5696	*ΔrfaH::kan* in WAM5678 (*ΔstpA* excised of *kan* marker), used in ChIP-seq as *"ΔstpAΔrfaH"*	*This study*
WAM5701	*ΔrfaH* excised of marker by pCP20 in 5678 (*ΔstpA* excised of kan marker)	*This study*
ΔhnsΔstpA	*Δhns::kan* freshly transduced into *ΔstpA* excised of kan marker (WAM5678) (used in ChIP-seq as *ΔhnsΔstpA)*	*This study*
ΔhnsΔstpAΔrfaH	*Δhns::kan* freshly transduced into *ΔrfaHΔstpA* excised of kan marker (WAM5701)*, used in ChIP-seq as "ΔhnsΔstpAΔrfaH"*	*This study*
WAM5086	*ΔstpA::kan* in WAM4507 generated by λ red recombineering	*This study*
WAM5087	*Δhns::kan* in WAM4507 generated by λ red recombineering	*This study*
WAM5686	*ΔrfaH::kan* in WAM4507 generated by λ red recombineering	*This study*
WAM5700	*ΔrfaH* excised of marker by pCP20 in WAM4505	*This study*
RL3000	MG1655 (F-, λ-, ilvG468+, rfb-50, rph+ ybhJ(L54->I) yebN(G25xxx->D) ycfK::97bp ΔInsB-5 ΔInsA-5 ΔInsAB-5)	(38)
RL3504	BL21 *Δhns* transformed with pWT_Nat_StpA (over-expression StpA ithout tags) and pHiC	*This study*

Supplemental References

1. Datsenko KA, Wanner BL. 2000. One-step inactivation of chromosomal genes in Escherichia coli K-12 using PCR products. Proc Natl Acad Sci U S A 97:6640-5.
2. Banerjee R, Weisenhorn E, Schwartz KJ, Myers KS, Glasner JD, Perna NT, Coon JJ, Welch RA, Kiley PJ. 2020. Tailoring a Global Iron Regulon to a Uropathogen. mBio 11.
3. Battaglioli EJ, Baisa GA, Weeks AE, Schroll RA, Hryckowian AJ, Welch RA. 2011. Isolation of generalized transducing bacteriophages for uropathogenic strains of Escherichia coli. Appl Environ Microbiol 77:6630-5.
4. Dame RT, Luijsterburg MS, Krin E, Bertin PN, Wagner R, Wuite GJ. 2005. DNA bridging: a property shared among H-NS-like proteins. J Bacteriol 187:1845-8.
5. Boudreau BA, Hron DR, Qin L, van der Valk RA, Kotlajich MV, Dame RT, Landick R. 2018. StpA and Hha stimulate pausing by RNA polymerase by promoting DNA-DNA bridging of H-NS filaments. Nucleic Acids Res 46:5525-5546.
6. Baba T, Ara T, Hasegawa M, Takai Y, Okumura Y, Baba M, Datsenko KA, Tomita M, Wanner BL, Mori H. 2006. Construction of Escherichia coli K-12 in-frame, single-gene knockout mutants: the Keio collection. Mol Syst Biol 2:2006 0008.
7. Sonnenfield JM, Burns CM, Higgins CF, Hinton JC. 2001. The nucleoid-associated protein StpA binds curved DNA, has a greater DNA binding affinity than H-NS and is present in significant levels in hns mutants. Biochimie 83:243-249.
8. Belogurov GA, Mooney RA, Svetlov V, Landick R, Artsimovitch I. 2009. Functional specialization of transcription elongation factors. EMBO J 28:112-22.
9. Peters JM, Mooney RA, Grass JA, Jessen ED, Tran F, Landick R. 2012. Rho and NusG suppress pervasive antisense transcription in Escherichia coli. Genes Dev 26:2621-33.
10. Myers KS, Yan H, Ong IM, Chung D, Liang K, Tran F, Keles S, Landick R, Kiley PJ. 2013. Genome-scale analysis of escherichia coli FNR reveals complex features of transcription factor binding. PLoS Genet 9:e1003565.
11. Neidhardt FC, Bloch PL, Smith DF. 1974. Culture Medium for Enterobacteria. 119:736-747.

12. Davis SE, Mooney RA, Kanin EI, Grass J, Landick R, Ansari AZ. 2011. Mapping E. coli RNA polymerase and associated transcription factors and identifying promoters genome-wide. Methods Enzymol 498:449-71.

13. Molder F, Jablonski KP, Letcher B, Hall MB, Tomkins-Tinch CH, Sochat V, Forster J, Lee S, Twardziok SO, Kanitz A, Wilm A, Holtgrewe M, Rahmann S, Nahnsen S, Koster J. 2021. Sustainable data analysis with Snakemake. F1000Res 10:33.

14. Sheffield NC, Stolarczyk M, Reuter VP, Rendeiro AF. 2021. Linking big biomedical datasets to modular analysis with Portable Encapsulated Projects. Gigascience 10.

15. Martin M. 2011. Cutadapt removes adapter sequences from high-throughput sequencing reads. 2011 17:3.

16. Langmead B, Salzberg SL. 2012. Fast gapped-read alignment with Bowtie 2. Nat Methods 9:357-9.

17. Kroner GM, Wolfe MB, Freddolino PL. 2019. Escherichia coli Lrp Regulates One-Third of the Genome via Direct, Cooperative, and Indirect Routes. J Bacteriol 201.

18. Ramirez F, Ryan DP, Gruning B, Bhardwaj V, Kilpert F, Richter AS, Heyne S, Dundar F, Manke T. 2016. deepTools2: a next generation web server for deep-sequencing data analysis. Nucleic Acids Res 44:W160-5.

19. Zhang Y, Liu T, Meyer CA, Eeckhoute J, Johnson DS, Bernstein BE, Nusbaum C, Myers RM, Brown M, Li W, Liu XS. 2008. Model-based analysis of ChIP-Seq (MACS). Genome Biol 9:R137.

20. Welch RA, Burland V, Plunkett G, 3rd, Redford P, Roesch P, Rasko D, Buckles EL, Liou SR, Boutin A, Hackett J, Stroud D, Mayhew GF, Rose DJ, Zhou S, Schwartz DC, Perna NT, Mobley HL, Donnenberg MS, Blattner FR. 2002. Extensive mosaic structure revealed by the complete genome sequence of uropathogenic Escherichia coli. Proc Natl Acad Sci U S A 99:17020-4.

21. Deatherage DE, Barrick JE. 2014. Identification of mutations in laboratory-evolved microbes from next-generation sequencing data using breseq. Methods Mol Biol 1151:165-88.

22. Decker KT, Gao Y, Rychel K, Al Bulushi T, Chauhan SM, Kim D, Cho BK, Palsson BO. 2022. proChIPdb: a chromatin immunoprecipitation database for prokaryotic organisms. Nucleic Acids Res 50:D1077-D1084.

23. Sastry AV, Gao Y, Szubin R, Hefner Y, Xu S, Kim D, Choudhary KS, Yang L, King ZA, Palsson BO. 2019. The Escherichia coli transcriptome mostly consists of independently regulated modules. Nat Commun 10:5536.

24. Tjaden B. 2020. A computational system for identifying operons based on RNA-seq data. Methods 176:62-70.

25. Mooney RA, Davis SE, Peters JM, Rowland JL, Ansari AZ, Landick R. 2009. Regulator trafficking on bacterial transcription units in vivo. Mol Cell 33:97-108.

26. Grigorova IL, Phleger NJ, Mutalik VK, Gross CA. 2006. Insights into transcriptional regulation and sigma competition from an equilibrium model of RNA polymerase binding to DNA. Proc Natl Acad Sci U S A 103:5332-7.

27. Bon M, McGowan SJ, Cook PR. 2006. Many expressed genes in bacteria and yeast are transcribed only once per cell cycle. FASEB J 20:1721-3.

28. Zhang Y, Mooney RA, Grass JA, Sivaramakrishnan P, Herman C, Landick R, Wang JD. 2014. DksA guards elongating RNA polymerase against ribosome-stalling-induced arrest. Mol Cell 53:766-78.

29. Miller JB, Pickett BD, Ridge PG. 2019. JustOrthologs: a fast, accurate and user-friendly ortholog identification algorithm. Bioinformatics 35:546-552.

30. Whiteside MD, Winsor GL, Laird MR, Brinkman FS. 2013. OrtholugeDB: a bacterial and archaeal orthology resource for improved comparative genomic analysis. Nucleic Acids Res 41:D366-76.

31. Edgar RC. 2004. MUSCLE: multiple sequence alignment with high accuracy and high throughput. Nucleic Acids Res 32:1792-7.

32. Shen BA, Hustmyer CM, Roston D, Wolfe MB, Landick R. 2022. Bacterial H-NS contacts DNA at the same irregularly spaced sites in both bridged and hemi-sequestered linear filaments. iScience 25:104429.

33. Robinson JT, Thorvaldsdottir H, Winckler W, Guttman M, Lander ES, Getz G, Mesirov JP. 2011. Integrative genomics viewer. Nat Biotechnol 29:24-6.

34. R Development Core Team. 2021. R: A language and environment for statistical computing., R Foundation for Statistical Computing, Vienna, Austrai. https://www.R-project.org/.

35. Ryan MC, Stucky M, Wakefield C, Melott JM, Akbani R, Weinstein JN, Broom BM. 2019. Interactive Clustered Heat Map Builder: An easy web-based tool for creating sophisticated clustered heat maps. F1000Res 8.

36. Tuckey C, Asahara H, Zhou Y, Chong S. 2014. Protein synthesis using a reconstituted cell-free system. Curr Protoc Mol Biol 108:16 31 1-22.

37. Mobley HL, Green DM, Trifillis AL, Johnson DE, Chippendale GR, Lockatell CV, Jones BD, Warren JW. 1990. Pyelonephritogenic Escherichia coli and killing of cultured human renal proximal tubular epithelial cells: role of hemolysin in some strains. Infect Immun 58:1281-9.

38. Ghosh IN, Landick R. 2016. OptSSeq: High-Throughput Sequencing Readout of Growth Enrichment Defines Optimal Gene Expression Elements for Homoethanologenesis. ACS Synth Biol 5:1519-1534.
